# Learning high-accuracy error decoding for quantum processors

**DOI:** 10.1038/s41586-024-08148-8

**Published:** 2024-11-20

**Authors:** Johannes Bausch, Andrew W. Senior, Francisco J. H. Heras, Thomas Edlich, Alex Davies, Michael Newman, Cody Jones, Kevin Satzinger, Murphy Yuezhen Niu, Sam Blackwell, George Holland, Dvir Kafri, Juan Atalaya, Craig Gidney, Demis Hassabis, Sergio Boixo, Hartmut Neven, Pushmeet Kohli

**Affiliations:** 1 Google DeepMind, London, UK; 2 Google Quantum AI, Santa Barbara, CA USA

**Keywords:** Computational science, Qubits, Information theory and computation

## Abstract

Building a large-scale quantum computer requires effective strategies to correct errors that inevitably arise in physical quantum systems^1^. Quantum error-correction codes^2^ present a way to reach this goal by encoding logical information redundantly into many physical qubits. A key challenge in implementing such codes is accurately decoding noisy syndrome information extracted from redundancy checks to obtain the correct encoded logical information. Here we develop a recurrent, transformer-based neural network that learns to decode the surface code, the leading quantum error-correction code^3^. Our decoder outperforms other state-of-the-art decoders on real-world data from Google’s Sycamore quantum processor for distance-3 and distance-5 surface codes^4^. On distances up to 11, the decoder maintains its advantage on simulated data with realistic noise including cross-talk and leakage, utilizing soft readouts and leakage information. After training on approximate synthetic data, the decoder adapts to the more complex, but unknown, underlying error distribution by training on a limited budget of experimental samples. Our work illustrates the ability of machine learning to go beyond human-designed algorithms by learning from data directly, highlighting machine learning as a strong contender for decoding in quantum computers.

## Abstract (web summary)

A recurrent, transformer-based neural network, called AlphaQubit, learns high-accuracy error decoding to suppress the errors that occur in quantum systems, opening the prospect of using neural-network decoders for real quantum hardware.

## Main

The idea that quantum computation has the potential for computational advantages over classical computation, both in terms of speed and resource consumption, dates all the way back to Feynman^5^. Beyond Shor’s well-known prime factoring algorithm^6^ and Grover’s quadratic speed-up for unstructured search^7^, many potential applications in fields such as material science^8^, machine learning^9^ and optimization^10^ have been proposed.

Yet, for practical quantum computation to become a reality, errors on the physical level of the device need to be corrected so that deep circuits can be run with high confidence in their result. Such fault-tolerant quantum computation can be achieved through redundancy introduced by combining multiple physical qubits into one logical qubit^1^. Ultimately, to perform fault-tolerant quantum computation such as the factorization of a 2,000-bit number, the logical error rate needs to be reduced to about 10^−12^ per logical operation^3,11^, far below the error rates in today’s hardware that are around 10^−3^ to 10^−2^ per physical operation.

**Fig. 1:**
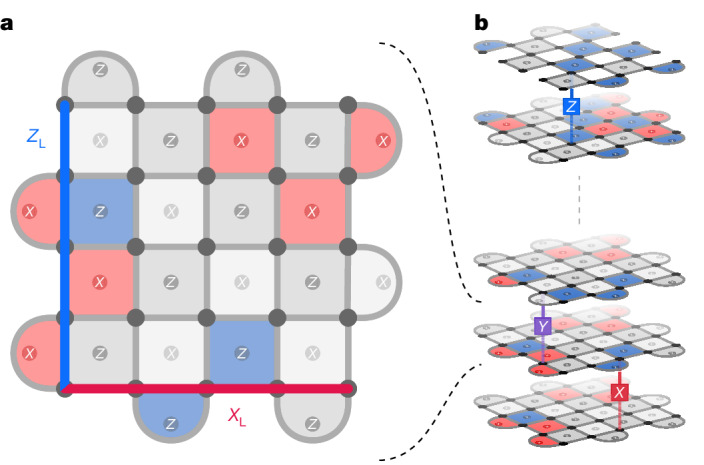
The rotated surface code and a memory experiment. **a**, Data qubits (grey circles) on a *d* × *d* square lattice (here shown for code distance *d* = 5) are interspersed with *X* and *Z* stabilizer qubits (*X* and *Z* in circles). The logical observables *X*_L_ (*Z*_L_) are defined as products of *X* (*Z*) operators along a row (column) of data qubits. **b**, In a memory experiment, a logical qubit is initialized, repeated stabilizer measurements are performed and then the logical qubit state is measured. During the experiment all qubits and operations are subject to errors (here symbolically shown as bit (*X*), phase (*Z*), and combined bit and phase flips (*Y*) acting on individual data qubits from time step to time step).

One of the most promising strategies for fault-tolerant computation is based on the surface code (Fig. 1), which has the highest-known tolerance for errors of any code with a planar connectivity^3,12,13^. In the surface code, a logical qubit is formed by a *d* × *d* grid of physical qubits, called data qubits, such that errors can be detected by periodically measuring *X* and *Z* stabilizer checks on groups of adjacent data qubits, using *d*^2^ − 1 stabilizer qubits located between the data qubits (Fig. 1a). A detection event (or event) occurs when two consecutive measurements of the same stabilizer give different parity outcomes. A pair of observables *X*_L_ and *Z*_L_, which commute with the stabilizers but anti-commute with each other, define the logical state of the surface code qubit. The minimum length of these observables is called the code distance, which represents the number of errors required to change the logical qubit without flipping a stabilizer check. In a square surface code, this is the side length *d* of the data-qubit grid.

The task of an error-correction decoder is to use the history of stabilizer measurements, the error syndrome, to apply a correction to the noisy logical measurement outcome to obtain the correct one. In the near term, highly accurate decoders can enable proof-of-principle demonstrations of fault tolerance. Longer term, they can boost the effective performance of the quantum device, requiring fewer physical qubits per logical qubit or reducing requirements on device accuracy^3,4,14^.

Quantum error correction frequently requires different decoding methods to classical error correction^15,16^ and, despite recent significant progress^4,17–21^, challenges remain. A quantum error-correction decoder must contend with complex noise effects that include leakage, that is, qubit excitations beyond the computational states $| 0\rangle$ and $| 1\rangle$ that are long-lived and mobile^22^; and cross-talk, that is, unwanted interactions between qubits inducing long-range and complicated patterns of events^23^. These effects fall outside the theoretical assumptions underlying most frequently used quantum error-correction decoders, such as minimum-weight perfect matching (MWPM)^16,24^. Extending decoders to account for the complex noise effects described above^4,25–27^ or suppressing them on the hardware side^23,28,29^ are areas of active research. A further challenge for error-correcting real-world quantum devices is the difficulty in modelling errors accurately^4,14,30,31^. In principle, a decoder that adapts to more realistic noise sources and that learns directly from data (without the need to fit precise noise models) can help to realize a fault-tolerant quantum computer using realistic noisy hardware.

## Machine-learning quantum error-correction decoders

Recently, there has been an explosion of machine-learning techniques applied to quantum computation, including decoding (Extended Data Table 1). Some previous studies have used supervised learning^32^ or reinforcement learning^33^ to train neural-network-based decoders. Most consider qubit-level (as opposed to circuit-level) errors, which greatly simplifies the decoding problem as all errors are local in time.

A handful of studies consider more realistic circuit-level noise models. Chamberland et al.^34^ trained recurrent and convolutional networks and matched look-up table performance for surface codes and colour codes with code distances up to 5. Baireuther et al.^32^ trained a long short-term memory (LSTM) decoder for a colour code corrupted by both Pauli and beyond-Pauli circuit-level noise. Zhang et al.^35^ developed fast decoders based on three-dimensional convolutions, and applied them to surface codes up to distance 7 with experimentally inspired circuit-level noise, but did not achieve MWPM performance.

More recently, two studies have tested the performance of machine-learning decoders using the Sycamore surface code experiment. Varbanov et al.^36^ built a recurrent-neural-network decoder based on the architecture of Baireuther et al.^32,37^. They trained it on a circuit-level depolarizing noise model (with noise parameters fitted to the experimental data) and evaluated on experimental data, approaching parity with the best previously published result at code distance 3. Furthermore, they quantified the benefits of modelling correlations and of using analogue inputs (modelled by a symmetric Gaussian I/Q—in-phase (I) and quadrature (Q)—readout noise model^38^), which yielded a slight increase in accuracy. Lange et al.^39^ trained a graph-neural-network decoder using both circuit-level and experimental data, showing parity with a standard MWPM decoder.

**Fig. 2:**
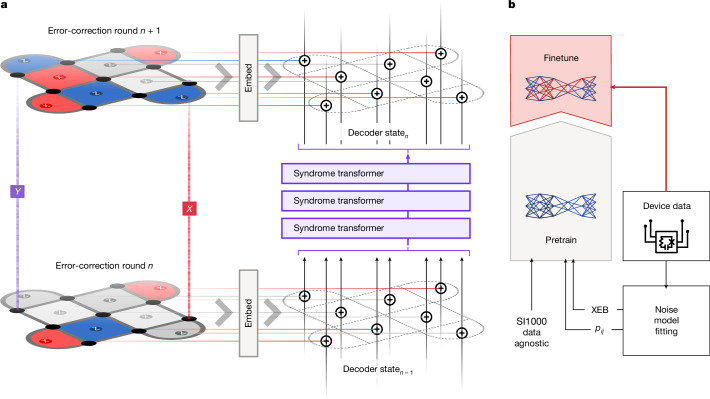
Error correction and training of AlphaQubit. **a**, One error-correction round in the surface code. The *X* and *Z* stabilizer information updates the decoder’s internal state, encoded by a vector for each stabilizer. The internal state is then modified by multiple layers of a syndrome transformer neural network containing attention and convolutions. The state at the end of an experiment is used to predict whether an error has occurred. **b**, Decoder training stages. Pretraining samples come either from a data-agnostic SI1000 noise model, or from an error model derived from experimental data using *p*_*ij*_ or XEB methods^4,31^.

In this work, we present AlphaQubit, a recurrent-transformer-based neural-network architecture that learns to predict errors in the logical observable based on the syndrome inputs (Methods and Fig. 2a). This network, after two-stage training—pretraining with simulated samples and finetuning with a limited quantity of experimental samples (Fig. 2b)—decodes the Sycamore surface code experiments more accurately than any previous decoder (machine learning or otherwise).

On simulated data, which model a near-term device with an approximately 6% detection event density (Fig. 4a, inset) using a richer noise model than those considered in previous machine-learning decoder work—including leakage, cross-talk and soft readouts with an amplitude damping component—AlphaQubit scales to code distance 11 and generalizes to 100,000 error-correction rounds while maintaining accuracy beyond correlated MWPM (MWPM-Corr). AlphaQubit benefits from analogue inputs (as previously observed^36^) and we show it maintains an accuracy lead against MWPM-Corr augmented to process analogue inputs^40^.

## Quantum error correction on current quantum devices

As physical error rates in quantum hardware have been brought down, researchers have started to conduct error-suppression experiments on real quantum devices^4,17,18,20,21,41^. We first apply AlphaQubit to Google’s Sycamore memory experiment^42^, which comprises both *X*-basis and *Z*-basis memory experiments on surface codes with distance 3 and distance 5. The 3 × 3 code block was executed at 4 separate locations on the Sycamore chip, and the 5 × 5 code block was executed at a single location. Fifty thousand experiments were performed for each total rounds count *n* ∈ {1, 3, …, 25}, and the resulting data were split into even and odd subsets for twofold cross-validation. Below we describe training on even with final testing on odd.

**Fig. 3:**
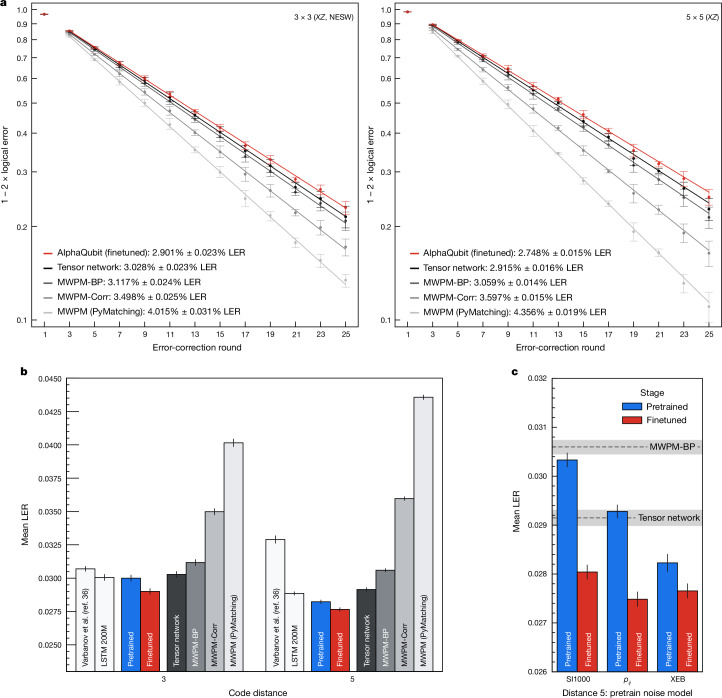
Logical error per round on the 3 × 3 and 5 × 5 Sycamore experiment. All AlphaQubit results (both pretrained and finetuned) are for ensembles of 20 models. All results are averaged across bases, even and odd cross-validation splits, and, for the 3 × 3 experiments, the location (north, east, south, west (NESW)), and are fitted across experiments of different durations. **a**, The 1 − 2 × logical error versus error-correction round for code distance-3 and distance-5 memory experiments in the Sycamore experimental dataset for the baseline tensor-network decoder (black), our decoder (red) and three variants of MWPM (shades of grey). The LER is calculated from the slope of the fitted lines. The error bars are the 95% confidence interval. **b**, LERs of our decoders and other published results for the Sycamore experiment data. We also show the performance of an LSTM model pretrained on XEB DEM data. Error bars are standard bootstrap errors. **c**, LERs of our decoder pretrained on different noise models, and after finetuning on experimental data. Error bars are standard bootstrap errors.

Decoder performance is quantified by the logical error per round (LER), the fraction of experiments in which the decoder fails for each additional error-correction round^4^ (‘Logical error per round’ in Methods and Fig. 3a).

Decoders are trained for a specific distance, basis and location, but can decode experiments of any number of rounds. Training is in two stages: pretraining and finetuning (‘Sycamore data’ in Methods and Fig. 2b). In the pretraining stage, we train on one of three kinds of simulated data with different degrees of similarity to the experimental data. In the first two scenarios, we pretrain on up to 2 billion samples drawn from detector error noise models (DEMs)^43^. The DEMs are either fitted to the (even) detection error event correlations *p*_*ij*_ (ref. ^4^) or use weights derived from a Pauli noise model that approximates the noise that occurs on hardware, based on device calibration data (from cross-entropy benchmarks (XEB); ‘Detector error model’ in Methods). In the third scenario, we pretrain on up to 500 million samples of superconducting-inspired circuit depolarizing noise (SI1000 noise^44^; ‘Circuit depolarizing noise’ in Methods), which does not depend on the experimental data or quantum device except in choosing the overall noise scale to approximately match experimental event densities.

For the finetuning stage, we partition the 325,000 even experimental samples into training and validation sets (‘Sycamore data’ in Methods). This procedure allows us to train a decoder to high accuracy with limited access to experimental data, while holding back the other fold (odd) as a test set.

AlphaQubit achieves an LER of (2.901 ± 0.023) × 10^−2^ at distance 3 and (2.748 ± 0.015) × 10^−2^ at distance 5 (Fig. 3a,b), giving an error-suppression ratio *Λ* = 1.056 ± 0.010, where ensembling 20 independently trained models contributes 0.03 × 10^−2^ (0.08 × 10^−2^) improvement at code distance 3 (5) (‘Ensembling’ in Methods). This LER is even lower than that of the tensor-network decoder—(3.028 ± 0.023) × 10^−2^ at distance 3, (2.915 ± 0.016) × 10^−2^ at distance 5 and *Λ* = 1.039 ± 0.010—to our knowledge, the most accurate decoder hitherto reported for this experiment^4,45^ but impractical for larger code distances owing to its computational cost. State-of-the-art MWPM-based decoders, such as correlated matching (MWPM-Corr), matching with belief propagation (MWPM-BP) and PyMatching, an open-source implementation of MWPM^4,24,26^, lead to higher LERs than the tensor network and AlphaQubit (Fig. 3a,b). For comparison, we also show the results of the LSTM-based neural network from Varbanov et al.^36^ and our own implementation of an LSTM (both pretrained on XEB DEMs). These achieve good results for 3 × 3. Varbanov’s LSTM-based neural network fails to match the tensor-network decoder at 5 × 5 (Fig. 3b). Although our LSTM achieves this, it does not scale to larger code distances (see next section).

Pretraining with samples from a noise model matched to the experimental data (*p*_*ij*_ or XEB DEMs) leads to better performance than using the device-agnostic SI1000 (Fig. 3c). The *p*_*ij*_ DEMs are the same noise model that set the prior for the matching-based and tensor-network decoders. On this prior, our decoder achieves parity with the tensor-network decoder (within error). We note that even when pretraining with SI1000 samples, and without any finetuning, AlphaQubit achieves parity with MWPM-BP at code distance 5.

Finetuning with a limited amount of experimental data decreases the LER gap between models pretrained with well-matched (*p*_*ij*_ and XEB) and general (SI1000) priors; and improves the LER of all models well beyond the tensor-network decoder (Fig. 3c).

## Quantum error correction for future quantum devices

### Simulating future quantum devices

To achieve reliable quantum computation, the decoder must scale to higher code distances. To assess the decoder’s accuracy on envisioned hardware with error rates significantly lower than the Sycamore experimental data (‘Training details’ in Methods and Extended Data Fig. 2b) and distances beyond 5, we explore the performance of our decoder on simulated data (in place of experimental samples) at code distances 3, 5, 7, 9 and 11 (17–241 physical qubits).

**Fig. 4:**
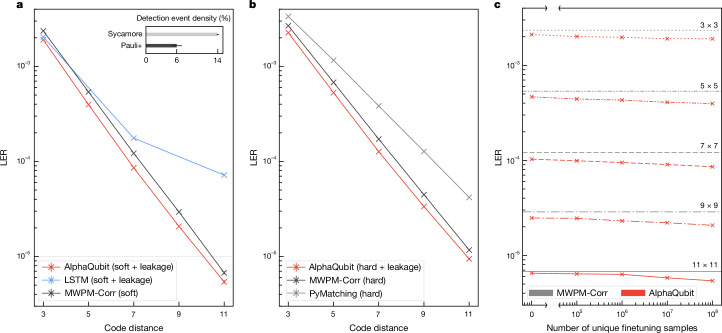
Larger code distances and finetuning accuracy trade-off. **a**,**b**, LER of different decoders for Pauli+ noise at different code distances. For each code distance, our decoder (red) is finetuned on 100 million samples from this noise model after pretraining on a device-agnostic circuit depolarizing noise model (SI1000). MWPM-Corr (black) and PyMatching (grey) are calibrated with a DEM tuned specifically to the Pauli+ noise model with soft information. The error bars are bootstrap standard errors. **a**, Soft decoder inputs. Inset: detection event density of the Pauli+ simulation compared with the Sycamore experimental samples (error bars are standard error of the mean). **b**, Hard decoder inputs. **c**, LER of AlphaQubit (soft inputs) pretrained on SI1000 noise and finetuned with different number of unique Pauli+ samples at code distances 3–11.

To go beyond conventional circuit noise models^37,46^ we use a Pauli+ simulator (‘Pauli+ model for simulations with leakage’ in Methods) that can model crucial real-world effects such as cross-talk and leakage. The simulator’s readouts are further augmented with soft I/Q information that models a dispersive measurement of superconducting qubits, to capture underlying analogue information about uncertainty and leakage^38,47,48^ (‘Measurement noise’ and ‘Simulating future quantum devices’ in Methods, Fig. 4a, inset, and Extended Data Fig. 2a). These analogue I/Q measurements and derived events are provided to AlphaQubit in the form of probabilities^40^ (‘Soft measurement inputs versus soft event inputs’ and ‘Input representation’ in Methods).

### Decoding at higher distances

For each code distance, we pretrain our decoder on up to 2.5 billion samples from a device-agnostic circuit depolarizing noise model (SI1000 with a simple variant of I/Q readout and leakage) before using a limited amount of data generated by the Pauli+ simulator (with realistic simulation of leakage and full I/Q noise; ‘Pauli+’ in Methods) to stand in as experimental data for finetuning. In Fig. 4a, we show the LER at each code distance after finetuning.

To establish strong baselines, we compare MWPM-Corr with a DEM tuned specifically for the Pauli+ noise model and augmented to benefit from analogue readouts. We also include our LSTM decoder, trained for code distances 3, 7 and 11 with unlimited Pauli+ training samples.

AlphaQubit achieves the highest accuracy for all code distances up to 11, surpassing even the correlated matching decoder augmented with soft information (Fig. 4a). At distance 3, the augmented MWPM-Corr LER is larger than the AlphaQubit LER by a factor of 1.25; by a factor of 1.4 at distance 9, and by a factor of 1.25 at distance 11.

We note that although the LSTM scales up to code distance 7, consistent with regimes tested in the literature^32,36^, it does not scale to distance 11 despite the significantly larger number of model parameters (200 million) compared with our model (5.4 million over all code distances; ‘Parameters’ in Methods).

For comparison, we also test our model on hard inputs (that is, where the analogue readouts were binarized before decoding). Although, as expected, both decoders perform worse, AlphaQubit maintains roughly the same improvement in error suppression compared with MWPM-Corr at distance 11 (LER ≈ 1.2 × 10^−5^ for MWPM-Corr versus LER ≈ 9 × 10^−6^ for AlphaQubit; Fig. 4b). When previous studies mention MWPM, they generally refer to its uncorrelated version^36,39,49^, which is weaker than MWPM-Corr, with an LER ≈ 4.1 × 10^−5^ at distance 11 (Fig. 4b).

To assess the effect of further limiting experimental data, at each code distance, we finetuned the same SI1000-pretrained base model using only 10^5^ to 10^8^ samples (Fig. 4c). As baselines, we show the corresponding MWPM-Corr performance from Fig. 4a, as well as the performance of the pretrained model before any finetuning. Despite the data-agnostic SI1000 prior, for code distances up to 11, the pretrained model is already on par with MWPM-Corr and further improves with more finetuning examples.

### Generalization to a streaming decoder

**Fig. 5:**
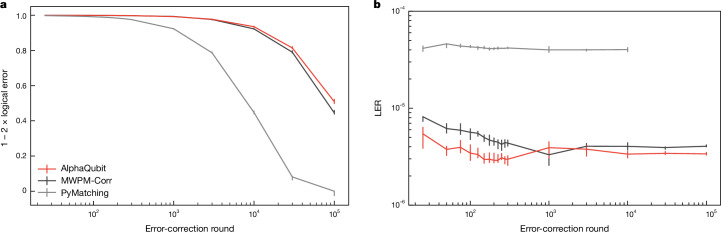
Generalization to larger number of error-correction rounds at code distance 11. **a**,**b**, The 1 − 2 × logical error after up to 100,000 error-correction rounds (**a**) and the corresponding LER (**b**) for PyMatching (grey), MWPM-Corr (black) and AlphaQubit (red) pretrained on SI1000 samples up to 25 rounds and finetuned on 10^8^ distance-11 Pauli+ simulated experiments of 25 rounds. Both finetuning and test samples are Pauli+. We plot LER values only where the corresponding 1 − 2 × logical error value is above 0.1. The error bars are bootstrap standard errors.

When training a decoder, the available pretraining and finetuning data will cover only a limited range of number of error-correction rounds. However, a practical decoder will need to perform equally well for longer experiments (‘Discussion and conclusion’, and ‘Generalization to logical computations’ in Methods). We demonstrate that AlphaQubit from the previous section, with its recurrent structure, can sustain its accuracy far beyond the 25 error-correction rounds that the decoder was trained on. We find its performance generalizes to experiments of at least 100,000 rounds (Fig. 5 and ‘Time scalability’ in Methods).

### Utility beyond error correction

**Fig. 6:**
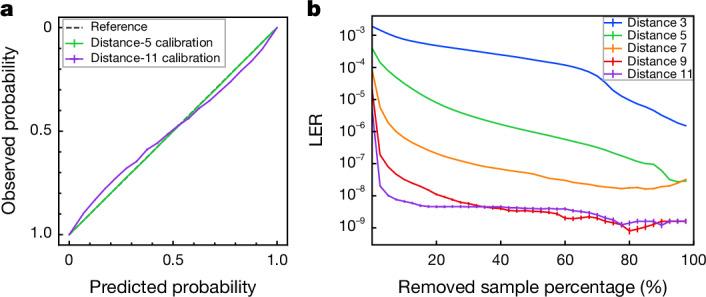
Using the network’s output as a confidence measure for post-selection. Calibration and post-selection data are evaluated on 10^9^ Pauli+ simulated experiments. **a**, Example calibration plot at distance 5 (green continuous line) and distance 11 (purple continuous line), with small but present error bars for s.e.m. The black dashed line represents a perfectly calibrated classifier. **b**, LER versus the fraction of low-confidence experiments discarded. Error bars are s.e.m. from values in each bin (visible for a LER ≲ 10^−8^).

As we trained the neural network by minimizing cross-entropy, its output can be interpreted as the probability of a logical error, a probability we found to be well calibrated (Fig. 6a and Extended Data Fig. 6a). For example, of samples with prediction probability 0.8, approximately 80% contain a logical error. Samples with a probability close to 0.5 are more likely to have been misclassified than samples with a probability closer to 0 or 1 (Extended Data Fig. 6b).

The probabilistic output can be used as soft information in hierarchical decoding schemes^50,51^, or as a confidence measure to discard the least confident samples (Fig. 6b). On Pauli+ simulated data, and by rejecting only 0.2% of the 25-round experiments at distance 11, we can reduce the error rate by a factor of about 20 (1% rejection gives a factor of about 107, 10% a factor of about 790), which can prove useful in protocols such as magic-state distillation, a major anticipated resource cost in fault-tolerant quantum computation^52,53^.

## Discussion and conclusion

We present AlphaQubit, a neural-network decoder designed to decode the surface code that can establish a state of the art in error suppression. On experimental data, it outperforms the previous best-in-class tensor-network decoder. Its accuracy persists at scale, continuing to outperform soft-input-augmented correlated matching at distances up to 11. AlphaQubit thus sets a benchmark for the field of machine-learning decoding, and opens up the prospect of using highly accurate machine-learning decoders in real quantum hardware.

Several challenges remain. Ultimately, to enable logical error rates below 10^−12^, we will need to operate at larger code distances. At distance 11, training appears more challenging (Fig. 4) and requires increasing amounts of data (Extended Data Fig. 7c). Although, in our experience, data efficiency can be markedly increased with training and architecture improvements, demonstrating high accuracy at distances beyond 11 remains an important step to be addressed in future work (‘Further considerations of scaling experiments’ in Methods).

Furthermore, decoders need to achieve a throughput of 1 μs per round for superconducting qubits^4,31^ and 1 ms for trapped-ion devices^20^. Improving throughput remains an important goal for both machine-learning and matching-based decoders^38,54–57^. Although the AlphaQubit throughput is slower than the 1-μs target (‘Decoding speed’ in Methods), a host of established techniques (‘Decoding speed’ in Methods) can be applied to speed it up, including knowledge distillation, lower-precision inference and weight pruning, as well as implementation in custom hardware.

To realize a fault-tolerant quantum computation, a decoder needs to handle logical computation. Graph-based decoders can achieve this through a windowing approach^58^. We envisage co-training network components, one for each gate needed for a logical circuit. To reduce complexity and training cost, these components might share parameters and be modulated by side inputs (such as gate type; ‘Generalization to logical computations’ in Methods). Such generalization abilities are intimated by our decoder’s generalization across rounds that far exceed its training regime and by the ability to train a single decoder to decode all of the code distances 3–11 of the scaling experiment (Fig. 4) with the same accuracy as the individual code-distance-trained decoders (Extended Data Fig. 7b). As our architecture is not specific to the surface code, we anticipate that it can be adapted to colour codes or other quantum low-density parity check codes.

As a machine-learning model, our decoder’s greatest strengths come from its ability to learn from real experimental data. This enables it to utilize rich inputs representing I/Q noise and leakage, without manual design of particular algorithms for each feature. This ability to use available experimental information showcases a strength of machine learning for solving scientific problems more generally.

Although we anticipate that other decoding techniques will continue to improve, this work supports our belief that machine-learning decoders may achieve the necessary error suppression and speed to enable practical quantum computing.

## Methods

### Learning to decode the surface code

In this work, we present a neural-network architecture that learns to decode the surface code under realistic hardware-level noise. The network combines a number of problem-specific features. Its per-stabilizer decoder state representation—a vector for each of the *d*^2^ − 1 stabilizers—stores information about the syndrome history up to the current round. Convolutions allow the spatial dissemination of information between adjacent stabilizer representations and at longer range when dilated. Self-attention allows the stabilizer state vectors to be updated based on the current state of each of the other stabilizers giving full interconnection with a limited number of parameters. The pooling and readout network aggregate information from the representations of the relevant stabilizers to make a logical error prediction.

Using experimental examples consisting of syndromes and the corresponding logical errors, we train the network to improve its predictions of the logical errors using backpropagation with a cross-entropy objective. The neural network processes the stabilizer readouts round by round with the syndrome transformer to iteratively update the decoder state representation (Fig. 2a). At the end of an experiment, the readout network uses the decoder state to predict whether a logical error occurred in the experiment (see ‘Model details’ for more details).

Because real experimental data are in limited supply, we train our decoder in a two-stage process (Fig. 2b). In a pretraining stage, we first prime the network based on samples from a generic noise model (such as circuit depolarizing noise, which describes errors in the device based on a Pauli error noise model) for which we can quickly generate as many samples as needed (for example, using a Clifford circuit simulator such as Stim^43^). In a finetuning stage, we optimize this model for a physical device by training on a limited quantity of experimental samples from the device.

With this two-stage training method, we achieve state-of-the-art decoding using current quantum hardware, and demonstrate the applicability of this decoder for larger-scale quantum devices. First, on experimental data from a Sycamore quantum processor on distance-3 and distance-5 surface codes^4^, when pretraining on synthetic data modelling device noise and then finetuning on experimental samples, we observe significantly better error suppression than state-of-the-art correlated matching and tensor-network decoders^4,26,27,31,45^. Second, anticipating larger future quantum devices, we demonstrate that our decoder achieves better accuracy than a correlated-matching-based decoder for code distances 3 to 11 using samples from a Pauli*+* quantum simulator modelling cross-talk, leakage and analogue readouts (in-phase (I) and quadrature (Q) signals^38^, I/Q for short). In this scenario, we again pretrain with a circuit-level depolarizing noise model before finetuning on samples from the Pauli+ simulator. In both scenarios, we can pretrain to high accuracy without experimental samples, and our two-stage training procedure further improves accuracy by finetuning with realistic amounts of experimental samples.

### Datasets and noise models

#### Memory experiments

A memory experiment is the most basic error-correction experiment to run, but is representative of the difficulty of fault-tolerant computation with the surface code^4^. We encode a known single (logical) qubit state $| {\psi }_{{\rm{i}}}\rangle$ as ${\rho }_{{\rm{i}}}=| {\psi }_{{\rm{i}}}\rangle \langle {\psi }_{{\rm{i}}}|$, perform multiple error-correction rounds (that is, stabilizer qubit measurements) and finally perform a (logical) measurement on the resulting state *ρ*_f_. We declare success if the decoded logical outcome matches the initial logical encoding.

In a real-world experiment, all operations on the physical qubits are noisy. From a theory perspective, various levels of abstraction in modelling real-world noise can be studied, for example, the noiseless case, the code capacity case (noiseless readouts) or the phenomenological case (noise on physical qubits and readouts). In this sense, phenomenological noise is the most realistic case. Yet beyond this qualitative classification of noise types to study, the actual noise model that is used to describe the various operations on the physical qubits can vary tremendously in accuracy, from simple bit- or phase-flip noise, to a full simulation of the master equation of the quantum system. (And even then, how accurately the master equations describe a real-world system can vary significantly).

#### The rotated surface code

**Extended Data Fig. 1:**
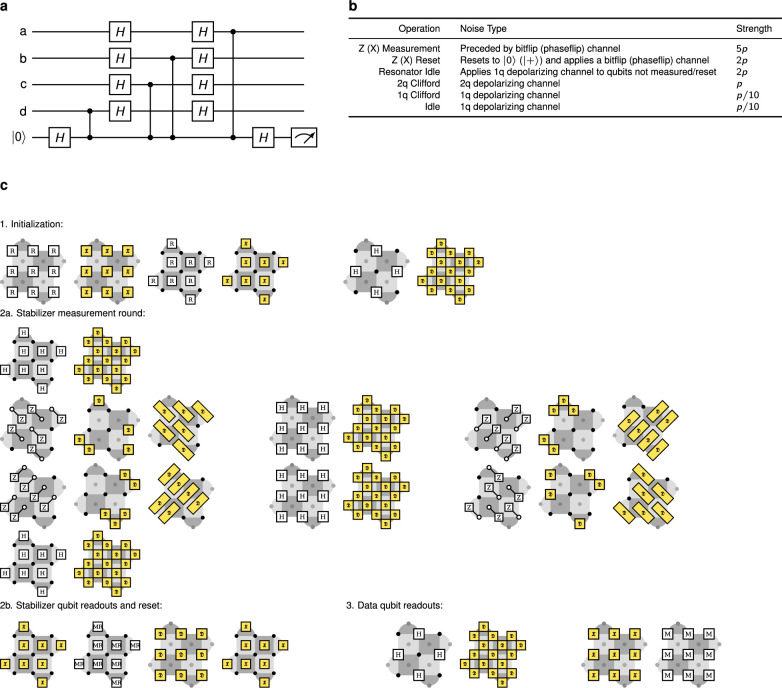
Stabilizer readout circuit for a **3 × 3** XZZX rotated surface code in the **Z** basis. (**a**) Common *X* and *Z* stabilizer readout for the XZZX code^61^. Here, the four first lines (a-d) are the data qubits surrounding a stabilizer qubit (last line), which has been reset to $| 0\rangle$. (**b**) Relative strength of noise operations in an SI1000 circuit depolarizing noise model, parameterized by *p*. (**c**) Corresponding circuit depolarizing noise gate and error schema. Black dots indicate data qubits, gray dots indicate *X*/*Z* stabilizer qubits, as detailed in Fig. 1a. ${\mathfrak{D}}$ (yellow blocks) labels single- or two-qubit depolarizing noise, and ${\mathfrak{X}}$ labels a bit flip channel. M, R, and MR are measurements, reset, and combined measurement and reset in the *Z* basis. H is a Hadamard gate, and CZ gates are indicated by their circuit symbol.

Here we study the memory experiment for a rotated surface code^59^, a variant of the surface code, which itself is a variant of Kitaev’s toric code^60^. In the rotated surface code, stabilizers are interspersed in a two-dimensional grid of data qubits (Fig. 1a). Stabilizer readouts are performed via stabilizer ancilla qubits, in a circuit as given in Extended Data Fig. 1. For all experiments, we use the *XZZX* circuit variants for the rotated surface code, which is Clifford-equivalent to the conventional CSS surface code^61^.

Although the *XZZX* code has the same stabilizers at every face (Extended Data Fig. 1), for visualization purposes we draw the usual CSS surface code where *X*-type and *Z*-type stabilizers are interleaved in a checkerboard-like pattern throughout the two-dimensional grid. Similarly, we denote those stabilizers that are collected in the first and final rounds of the memory experiments (that is, those that can be inferred from the initially prepared eigenstates and final measurements (Fig. 1b and Extended Data Fig. 4) as ‘on-basis’, whereas ‘off-basis’ refers to the stabilizers that cannot; so, for instance, in an *X*-basis memory experiment, the on-basis stabilizers would be the transformed *X*-type stabilizers from the usual CSS surface code.

#### Sycamore memory experiment dataset

This is the publicly released dataset^42^ accompanying Google’s Sycamore surface code experiment^4^, comprising:

1. Four areas at code size 3 × 3, dubbed north, south, east, and west; as well as one area for code size 5 × 5.
2. For each of the five areas: both *X* and *Z* memory experiment bases, which set the basis in which the logical qubit is initialized (randomly in $| +\rangle$ or $| -\rangle$ for an *X* experiment, and $| 0\rangle$ or $| 1\rangle$ for a *Z* experiment), and in which it is measured at the end.
3. For each of the five areas and two bases: individual experiments at 1, 3, 5, …, 25 error-correction rounds, at 50,000 shots each.

This means that 50,000 shots × 13 round settings × 2 bases × 5 areas = 6.5 × 10^6^ individual shots were recorded in total. Each dataset was split into an even and odd subset for twofold cross-validation, and accompanied by a DEM fitted to the respective subset, to be used for decoding the other fold, respectively.

#### Detector error model

A DEM^43^ can be thought of as an error hypergraph, where stochastic error mechanisms are hyperedges connecting the clusters of detectors they trigger. These mechanisms are independent and have an associated error probability. The DEMs we use were previously fitted^4^ to each experimental set using a generalization of the *p*_*ij*_ method^14^.

The XEB DEMs are computed by running a standard calibration suite that measures the fidelity of different gates (frequently using XEB benchmarking to compute the error rate, hence the name).

We use the open-source program Stim^43^ to generate samples using the DEMs. This is necessary for pretraining AlphaQubit, as the limited quantity of experimental data available makes training with only experimental data unfeasible (see ‘Training details’).

#### Circuit depolarizing noise

As error syndromes cannot be read out directly with a single measurement, a stabilizer readout circuit has to be applied to deduce the stabilizers, as shown in Extended Data Fig. 1. The entire sequence of circuit depolarizing noise for a memory experiment of the surface code is shown in Extended Data Fig. 1 for an *XZZX* circuit variant of the rotated surface code^61^.

SI1000 (superconducting-inspired 1,000-ns round duration) noise^44^ is a circuit depolarizing noise model comprising Pauli errors of non-uniform strengths, which approximate the relative noisiness of the various circuit processes in superconducting circuits: for example, as measurements remain a major source of errors in superconductors, measurement noise has weight 5*p* for noise parameter *p*. In contrast, single qubit gates and idling introduce only a small amount of noise, hence their relative strength is *p*/10.

#### Intermediate data

For some simulated data modalities, such as our SI1000 Stim-simulated data, we have enough privileged information about the quantum state to determine what would happen if we had finished the experiment earlier. For an experiment with *n* rounds, we can provide the result of data-qubit measurements, and consequently alternative ‘last rounds’ of detectors and logical observables, if they were to happen after rounds 1, 2, …, *n* − 1 instead of at the end of the experiment.

The no-cloning theorem makes these intermediate measurements not accessible in an experimental setting, so we do not use it as an input in our decoders. However, in simulated data, they provide an auxiliary label per experimental round, improving training by providing more information per sample.

#### Measurement noise

In each error-correction round, we projectively measure many of the qubits, allowing us to extract information about errors that have occurred. Consider measuring a self-adjoint operator *A* with discrete eigenvalues {*λ*_*i*_}_*i*__∈*I*_ for some index set *I*. Let *P*_*i*_ be the projector into the subspace with eigenvalue *λ*_*i*_. Then the probability of observing *λ*_*i*_ in a measurement is given by Born’s rule, *p*_*i*_ = Tr(*ρP*_*i*_), and in that case the resulting state is projected into *P*_*i*_*ρP*_*i*_/*p*_*i*_. For the case of a single qubit measured in the computational basis $\{| 0\rangle ,| 1\rangle \}$, ${p}_{| 0\rangle }=\langle 0| \rho | 0\rangle$ and ${p}_{| 1\rangle }=\langle 1| \rho | 1\rangle$. In an error-correction round, we only measure a subset of the qubits, but the projective nature of the measurement causes the entangled state of the data qubits to remain an eigenstate of all the stabilizer operators.

In practice, measurement is a challenging engineering problem: ordinarily, we want qubits isolated from their environment to allow coherent operations, but measurement necessitates interaction with the environment. In addition, we need to immediately re-use measured qubits for the next error-correction round, which requires either a ‘non-demolition’ measurement (where the qubit is faithfully projected into $| 0\rangle$ or $| 1\rangle$ corresponding to the measurement outcome) or other state preparation, such as unconditional reset to $| 0\rangle$ following measurement. Unconditional reset also provides an opportunity to remove leakage from the system^28^. Measurement can cause other problems such as state transitions and unwanted dephasing, which must be carefully avoided^62,63^.

Implementations vary between physical platforms. For example, in standard dispersive measurement of superconducting qubits, a linear resonator (or series of resonators) serves as an intermediary between the qubit and the outside world^47^. The measurement is implemented as a microwave scattering experiment to probe the resonator’s frequency, which depends on the qubit state due to coupling with nonlinear Josephson elements^48^. The scattered microwave pulse is amplified and digitized to determine its amplitude and phase, which encodes information about the qubit state^38^.

The resulting amplitude and phase is traditionally represented in a two-dimensional space of in-phase (I) and quadrature (Q) amplitudes, (I, Q). Ideally, there is a distinct point in (I, Q) space associated with each qubit state ($| 0\rangle$, $| 1\rangle$, and potentially leakage states such as $| 2\rangle$). However, the measured signals are obfuscated with noise from sources such as transmission loss, amplifiers and electronics, manifesting as a spread or ‘cloud’ of points in (I, Q) space associated with each state. In addition, qubits can show unwanted transitions between states during the measurement, such as decaying from $| 1\rangle$ to $| 0\rangle$, which would result in an average point between the $| 0\rangle$ and $| 1\rangle$ centres in (I, Q) space^64^. Ordinarily, a measured (I, Q) value is classified to $| 0\rangle$ or $| 1\rangle$ (or in some cases $| 2\rangle$), and this discrete measurement outcome is given to the decoder. However, a neural-network decoder can use the raw (I, Q) value instead, giving it access to richer information without further preprocessing.

**Extended Data Fig. 2:**
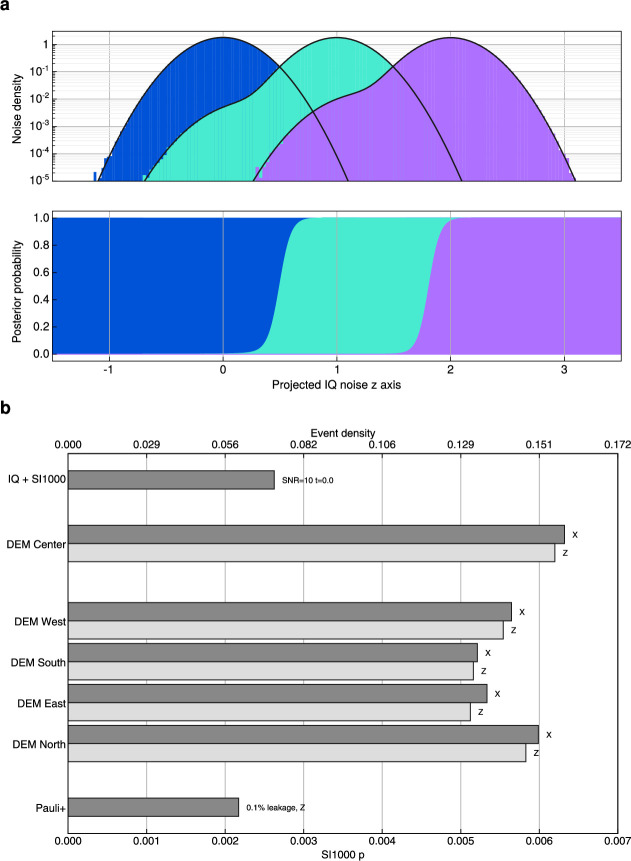
Noise and event densities for datasets used. (**a**) Simplified I/Q noise with signal-to-noise ratio SNR = 10 and normalised measurement time *t* = 0.01. Top plot: point spread functions when projected from its in-phase, quadrature, and time components onto a one-dimensional *z* axis. Shown are the sampled point spread functions for $| 0\rangle$ (blue), $| 1\rangle$ (green), and a leaked higher-excited state $| 2\rangle$ (violet). Bottom plot: posterior sampling probability for the three measurement states, for prior weights *w*_2_ = 0.5%, *w*_0_ = *w*_1_ = 49.75%. (**b**) Event densities for different datasets and the corresponding SI1000 p-value. We indicate the event density of the different datasets in the top x-axis, with a non-linear scale. The detector error models are fitted to the Sycamore surface code experiment^4^. All datasets use the XZZX circuit variant of the surface code (with CZ gates for the stabilizer readout, ‘The rotated surface code’ in Methods, Extended data Fig. 1). As we are never compiling between gatesets, there is no implied noise overhead; these are the final event densities observed when sampling from the respective datasets. For datasets with soft I/Q noise, the plots above show the average *soft* event density as explained in ‘Measurement noise’ in Methods.

To simulate this process, we run a simulation with noiseless measurements and then add noise after the fact. This can be as simple as discrete assignment error (for example, flip each measurement outcome with some probability) or we can emulate the richer (I, Q) signals. For our simulations, we consider a simplified one-dimensional space for our analogue readout signal, with probability density functions *P*_*i*_ for $| 0\rangle$, $| 1\rangle$ and $| 2\rangle$ centred around *z* = 0, 1 and 2, respectively, shown in Extended Data Fig. 2a. Although we could also consider higher-order leaked states (for example, by centring $| 3\rangle$ around 3, as for the other states) and present those to the network as separate inputs in a suitable fashion (for example, analogous to what we will describe below), and although they are produced by the Pauli+ simulation, we omit them in this analysis as they will be produced with even lower frequency than state $| 2\rangle$. For this reason, we map higher-order leaked states from the Pauli+ simulation to $| 2\rangle$, that is, we simply bucket states into ‘leaked’ ($| 2\rangle$) and ‘not leaked’ ($| 0\rangle$ and $| 1\rangle$).

These probability distributions are parameterized by a signal-to-noise ratio (SNR) and a dimensionless measurement duration, *t* = *t*_meas_/*T*_1_, the ratio of the measurement duration to the qubit lifetime, *T*_1_. The distribution for $| 0\rangle$, *P*_0_(*z*, SNR), is simply a Gaussian distribution centred at *z* = 0. For $| 1\rangle$, we centre at *z* = 1 and add the effect of decay from $| 1\rangle$ to $| 0\rangle$. For $| 2\rangle$, we centre at *z* = 2 and assume that the decay from $| 2\rangle$ to $| 1\rangle$ occurs roughly twice as frequently as the $| 1\rangle$ to $| 0\rangle$ case (in reality, qubits can deviate from this based on details in the qubit *T*_1_ spectra). For simplicity, we do not include the second-order process of decaying from $| 2\rangle$ to $| 1\rangle$ to $| 0\rangle$, although that does happen experimentally.

In this simplified single-parameter picture^40^ (see ‘Datasets and noise models’), we can thus write

$$\begin{array}{c}\,\,{P}_{0}(z,\,\text{SNR})=\sqrt{\frac{\,\text{SNR}\,}{\pi }}\exp (\,-\,\text{SNR}\times {z}^{2})\\ \,{P}_{1}(z,\,\text{SNR},\,t)=\frac{t}{2}\,\exp \,\left(-t\,(z-\frac{t}{4\,\text{SNR}\,})\right)\\ \,\,\,\,\,\,\,\times \,[\,\text{Erf}\,\left(\sqrt{\text{SNR}\,}(z-\frac{t}{2\,\text{SNR}})\right)\\ \,\,\,\,\,\,\,+\,\text{Erf}\,\left(\sqrt{\text{SNR}\,}(1-z+\frac{t}{2\,\text{SNR}})\right)]\\ \,\,\,\,\,\,\,+\,{{\rm{e}}}^{-t}\sqrt{\frac{\,\text{SNR}\,}{\pi }}\exp (\,-\,\text{SNR}\,{(z-1)}^{2})\\ {P}_{2}(z,\,\text{SNR},\,t)={P}_{1}(z-1,\,\text{SNR},\,2t).\end{array}$$

For each measurement outcome from the simulation of state $| i\rangle$, we sample an ‘observed’ value of *z* according to the associated probability density function. This ‘observed’ *z* can then be processed using a prior probability distribution and the known probability density functions to determine a posterior probability for each state. For example, we may have a prior distribution that leakage occurs with probability 0.01 and we split the remaining 0.99 evenly between $| 0\rangle$ and $| 1\rangle$. More generally, we express these posterior probabilities as

$$\begin{array}{l}{{\rm{post}}}_{1}:= {\rm{Prob}}\left(| 1\rangle \,| \,\neg | 2\rangle \right)\\ =\,\frac{{\rm{Prob}}(| 1\rangle \wedge \neg | 2\rangle )}{{\rm{Prob}}(\neg | 2\rangle )}=\frac{{\rm{Prob}}(| 1\rangle )}{{\rm{Prob}}(\neg | 2\rangle )}=\frac{{P}_{1}(z,\,{\rm{SNR}})}{({\bar{w}}_{0}/{\bar{w}}_{1}){P}_{0}(z,\,{\rm{SNR}})+{P}_{1}(z,\,{\rm{SNR}},\,t)}\end{array}$$

and

$${{\rm{post}}}_{2}:= {\rm{Prob}}(| 2)=\rangle \frac{{P}_{2}(z,\,{\rm{SNR}},t)}{{w}_{0}/{w}_{2}{P}_{0}(z,{\rm{SNR}})+{w}_{1}/{w}_{2}{P}_{1}(z,{\rm{SNR}},t)+{P}_{2}(z,{\rm{SNR}},\,t)},$$

where *w*_0_ + *w*_1_ + *w*_2_ = 1 are the prior probabilities of the three measurement outcomes, and ${\bar{w}}_{0}:= {w}_{0}/({w}_{0}+{w}_{1}),{\bar{w}}_{1}:= {w}_{1}/({w}_{0}+{w}_{1})$ are the marginal prior probabilities, conditioned on not having seen leakage.

This means we provide the network with two inputs:

1. post_1_: the posterior probability of observing a $| 1\rangle$ state, conditioned that the state was not leaked (that is, not in $| 2\rangle$). Once thresholded, this is the traditional measurement output from which syndrome or data-qubit measurements, and subsequent detection events, are derived.
  It is noted that owing to our ordering of the states $| 0\rangle$, $| 1\rangle$ and $| 2\rangle$ along the *z* axis (Extended Data Fig. 2a), if a state was leaked, it is most likely attributed to $| 1\rangle$, which is a valid choice of mapping an observed leaked state to a $| 0\rangle$ or $| 1\rangle$ measurement outcome. For matching-based decoders, this assignment is a valid choice of assignment of a leaked state to the $\{| 0,\rangle | 1\rangle \}$ subspace, and as good as, for example, a random mapping. Indeed, for a decoder unable to process leakage information, a leaked state is ‘lost information’, and thus an assignment to $| 1\rangle$ will create a detection event in about 50% of cases. (In the same fashion, if we were to include higher-order leaked states, attributing $| 3\rangle$, $| 4\rangle$ and so on to $| 1\rangle$ remains a valid choice).
2. post_2_: this is the probability of having seen leakage. Owing to the low prior probability of seeing leakage in first place (usually <1%), the posterior distributions are skewed against $| 2\rangle$, as can be seen in Extended Data Fig. 2a: even though the distribution for the state $| 2\rangle$ is centred around *z* = 2, has the same width as the other two distributions and additionally has a higher decay tail towards *z* = 1 owing to its twice-as-high normalized measurement time *t*, the prior weight shifts its posterior to only give a significant chance of interpreting a measurement outcome as $| 2\rangle$ at a *z* value already very close to *z* = 2.

#### Soft measurement inputs versus soft event inputs

For our model, we have found that directly providing stabilizer measurements as inputs instead of stabilizer detection events is beneficial (Extended Data Fig. 9). Traditionally, we have binary stabilizer readouts *s*_*i*,*n*_ ∈ {0, 1}, where *i* indexes the stabilizer qubit in the surface code and *n* indexes the error-correction round. A detection event is then derived simply as the change of a stabilizer measurement across error-correction rounds, *d*_*i*,*n*_ := *s*_*i*,*n*_ ⊕ *s*_*i*,*n*−1_, which itself is a binary variable ∈ {0, 1}. This is the quantity that is traditionally used by most decoders, for example, MWPM.

The XOR operation used to compute the change in stabilizer measurements results in a 1:1 correspondence of information encapsulated in the events input versus the measurements input; indeed, given detection events *d*_*i*,*n*_, we can—up to a possibly unknown initial measurement frame—obtain back the stabilizer measurements ${s}_{i,n}\equiv {\sum }_{m=0}^{n}{d}_{i,n}\,({\rm{mod}}\,\,2)$, where *d*_*i*,0_ is the first event frame. This first event frame was derived by either XOR’ing with an assumed zero frame before the first measurement (for example, for those stabilizers corresponding to the memory experiment basis; blue zeros in first stabilizer plot of Extended Data Fig. 4b), or was set to zero to remove an initially random stabilizer frame that does not allow extraction of more information about a first detection event (for example, for the off-basis stabilizers orthogonal to the memory experiment basis; see ‘The rotated surface code’).

This bijection allows us to present a comparison of measurement and event inputs, as they both contain the same amount of information for the decoder (possibly up to the first frame, as aforementioned; however, for Pauli noise, we take the initial off-basis stabilizers and XOR them onto the stabilizers anyhow, so that this assumed initial random frame is precisely zero as well).

If the measurements are transformed into posterior probabilities for each stabilizer measurement, we can assume that each such posterior

1 $${p}_{i,n}:= {{\rm{post}}}_{1}(i,n)={\rm{Prob}}({| 1\rangle }_{i,n}\,| \,{\rm{\neg }}{| 2\rangle }_{i,n})$$

parameterizes a Bernoulli random variable *M*_*i*,*n*_ ~ Ber(*p*_*i*,*n*_). As those are also Boolean-valued random quantities, we can then transform pairs of measurement variables into corresponding detection events, *E*_*i*,*n*_ ≔ *M*_*i*,*n*_ ⊕ *M*_*i*,*n*−1_, completely analogous to the binary measurement case. This means that

$${E}_{i,n}\sim {\rm{B}}{\rm{e}}{\rm{r}}({q}_{i,n})\,\text{where}\,{q}_{i,n}:={p}_{i,n}(1-{p}_{i,n-1})+(1-{p}_{i,n}){p}_{i,n-1}$$

is parameterized by the probability that exactly one of *M*_*i*,*n*_ and *M*_*i*,*n*−1_ is 1.

Analogously to before, this ‘soft XOR’ defines a linear recurrence on the detection events that can be integrated to obtain back the posterior measurement probabilities from the soft detection events:

$${p}_{i,-1}=0\,{\rm{and}}\,{p}_{i,n}=\frac{{q}_{i,n}-{p}_{i,n-1}}{1-2{p}_{i,n-1}}$$

It is clear from the above that the special case of complete uncertainty (*p*_*i*,*n*_ = 1/2 for some *n*) is non-invertible, as all information is lost in that case.

By induction, one can also show that for a series of measurements (for example, along an edge of data qubits in the surface code grid), thresholding the measurements against 1/2 and then XOR’ing the set is equivalent to performing an iterative ‘soft XOR’, and then thresholding. To show this, let us simplify notation and drop the multiindex; our soft measurement probabilities are *p*_1_, …, *p*_*n*_ such that all *p*_*i*_ ≠ 1/2, and the corresponding thresholded Boolean values are *z*_*i*_ := *p*_*i*_ > 1/2. We denote with SoftXOR(*p*_1_, …, *p*_*n*_) the soft XOR defined above, and want to show SoftXOR(*p*_1_, …, *p*_*n*_) > 1/2 if and only if (iff) *z*_1_ ⊕ … ⊕ *z*_*n*_. The induction start is then immediate from the definition, as SoftXOR(*p*_1_) = *p*_1_. Let us thus assume the hypothesis holds up to some value *m* − 1. Then

$$\begin{array}{c}{\rm{S}}{\rm{o}}{\rm{f}}{\rm{t}}{\rm{X}}{\rm{O}}{\rm{R}}({p}_{1},\ldots ,{p}_{m})={p}_{m}[1-{\rm{S}}{\rm{o}}{\rm{f}}{\rm{t}}{\rm{X}}{\rm{O}}{\rm{R}}({p}_{1},\ldots ,{p}_{m-1})]\\ \,\,\,\,\,\,\,\,\,+\,(1-{p}_{m}){\rm{S}}{\rm{o}}{\rm{f}}{\rm{t}}{\rm{X}}{\rm{O}}{\rm{R}}({p}_{1},\ldots ,{p}_{m-1})\\ \,\,\,\,\,\,\,\,\,=:\,{p}_{m}(1-b)+(1-{p}_{m})b\\ \,\,\,\,\,\,\,\,\, > 1/2\,\,{\rm{i}}{\rm{f}}\,{\rm{a}}{\rm{n}}{\rm{d}}\,{\rm{o}}{\rm{n}}{\rm{l}}{\rm{y}}\,{\rm{i}}{\rm{f}}\,b(1-2{p}_{m}) > \frac{1}{2}-{p}_{m}.\end{array}$$

Now if *p*_*m*_ < 1/2, we have 1 − 2*p*_*m*_ > 0 and thus *b* > 1/2; otherwise if *p*_*m*_ > 1/2, we have *b* < 1/2. Thus

$${\rm{S}}{\rm{o}}{\rm{f}}{\rm{t}}{\rm{X}}{\rm{O}}{\rm{R}}({p}_{1},\ldots ,{p}_{m}) > 1/2\,{\rm{i}}{\rm{f}}{\rm{f}}\,\left(b < \frac{1}{2}\vee {p}_{m} > \frac{1}{2}\right)\wedge \left(b > \frac{1}{2}\vee {p}_{m} < \frac{1}{2}\right).$$

The two cases then translate to

$$\begin{array}{rcl} &  & b < \frac{1}{2}\wedge {p}_{m} > \frac{1}{2}\quad {\rm{iff}}\quad \neg ({z}_{1}\oplus \ldots \oplus {z}_{m-1})\wedge {z}_{m}\,=: \,A\\  &  & b > \frac{1}{2}\wedge {p}_{m} < \frac{1}{2}\quad {\rm{iff}}\quad {z}_{1}\oplus \ldots \oplus {z}_{m-1}\wedge \neg {z}_{m}\,:= \,B,\end{array}$$

and *A* ∨ *B* = *z*_1_ ⊕ … ⊕ *z*_*m*_.

#### Pitfalls for training on soft information

A crucial safeguard in all machine-learning models is to never leak the label (that is, the value to be predicted) into the input of the model. For a distance-*d* rotated-surface-code experiment with binary stabilizer labels, there exist exactly *d*^2^ − 1 bits of information that are extracted at each error-correction round; and it is impossible to deduce, from these measurements alone, the logical state of the qubit in the experiment basis.

Naturally, this also holds true in the final round of a memory experiment, when we measure all data qubits in the experiment’s basis—*d*^2^ bits—and compute the on-basis stabilizer measurements from them—(*d*^2^ − 1)/2 many for an odd-distance surface code patch. As those stabilizers are a strict subset of the full *d*^2^ − 1 stabilizers derived in previous rounds, the same argument applies: no information about the logical state of the surface code qubit can be leaked, as all stabilizers commute with the logical operators of the code.

However, when reading *d*^2^ data qubits with soft information, and then re-computing the stabilizers from them via SoftXOR, there is a map of *d*^2^ floating point values to *d*^2^ − 1 floating point values. We found that this gives the model the ability to deduce the current logical state from the inputs, which poses an issue if the initial qubit state is not randomized. An intuition for why this might happen is that the model learns to (partially) invert the quadratic SoftXOR equations that map soft data-qubit measurements to the stabilizers, from which it can then learn what the logical observable should be; even if this inversion is not perfect, it is conceivable that the network might learn to extract a non-zero amount of additional information about the label, which should not have leaked into the inputs. For this reason, we always threshold the data-qubit measurements used for computing stabilizer measurements in the last memory experiment round (and the leakage data as well), irrespective of whether we were providing the model with soft or hard inputs in previous rounds. In this way, we ensure that there is precisely the same amount of information derived from the data qubits as in a standard ‘non-soft readouts’ memory experiment, that is, *d*^2^ bits, which in turn are mapped to (*d*^2^ − 1)/2 stabilizer measurements. This makes it impossible for any decoder to discern the logical measurement label from its inputs.

#### Pauli+ model for simulations with leakage

Realistic device noise was implemented in a manner similar to the Pauli+ model described in the supplementary material of the Sycamore experiment article^4^. This model was updated by scaling noise strengths down from the near-threshold regime in that work to realize approximately *Λ* = 4 in surface code performance for MWPM-Corr, where *Λ* is the ratio of logical error rates between two surface codes of distance *d* and *d* + 2, as in the supplementary material of ref. ^65^. Moreover, the simulator was modified from the ‘stabilizer tableau’ representation to use the Pauli-frame representation, which yields indistinguishable results as transitions between stabilizer states are Pauli channels.

We briefly review the Pauli+ model here, although details are described in the supplementary material of the Sycamore experiment article^4^. The Pauli+ model extends a Pauli-frame simulator with leakage states. These include transitions to and from leaked states, as well as error channels where a two-qubit gate applied to a qubit pair where one input is leaked is replaced by a noise channel on the non-leaked qubit. For a noise channel in the simulation (in general, a Kraus channel), the qubit subspace is Pauli twirled, and transitions to and from leaked states are converted to stochastic transitions. A Pauli-frame simulator is extended such that in addition to a Pauli operator at each qubit, leaked states can be tracked. For example, the possible states of one qubit with leaked excited states could be {*I*, *X*, *Y*, *Z*, L2, L3}, where L2 and L3 are states of the Pauli-frame simulator that represent quantum states $| 2\rangle$ and $| 3\rangle$.

The noise strength is adjusted to what might be achievable in superconducting quantum processors in the medium term, several years from the time of this study. Each gate in the simulation is associated with a baseline amount of depolarizing noise. The strength of depolarizing noise for each operation was informed by recent device characterization^4^ and an estimate of how noise might improve in future devices^65^. In addition to this conventional Pauli-channel noise, there is a model for coherent cross-talk that accounts for interactions between pairs of CZ gates; this model is Pauli twirled to produce Pauli channels that are correlated on groups of qubits up to size four, and the unitary calculated includes leakage levels^4^. Leakage is introduced in three ways. There is a probability of leakage introduced by dephasing during the CZ gate, a ‘heating rate’ of leakage that is a function of gate duration, and leakage terms that arise from the cross-talk unitary described above. The leakage rates were adjusted from the values in the supplementary material of the Sycamore experiment article^4^ such that CZ dephasing and cross-talk were reduced to 25% of the previous values (for example, CZ dephasing was 2 × 10^−4^ instead of 8 × 10^−4^), but the heating rate was unchanged at 1/(700 μs). When leakage is scaled in this work, it is these three rates that are scaled together. Leakage is removed from the system by multi-level reset gates applied after measurement, by data-qubit leakage removal^65^ applied to code qubits every syndrome round, and by a passive decay rate that is proportional to 1/*T*_1_.

#### Simulating future quantum devices

We use the Pauli+ simulator described in ‘Pauli+ model for simulations with leakage’ that can model and modulate the effects of cross-talk and leakage, and augment it with soft I/Q readouts, as described in ‘Measurement noise’, to generate data in place of experimental samples at code distances 3, 5, 7, 9 and 11. The density of detection events for our Pauli+ simulated data is roughly 60% lower than for the Sycamore experiment^4^ (Fig. 4a, inset, and Extended Data Fig. 2b) and about 0.1% of stabilizer measurements will appear leaked. For comparison, in the ‘Quantum error correction on current quantum devices’ section, each decoder is finetuned on 3.25 × 10^5^ samples; the entire experiment comprised 6.5 × 10^6^ unique shots in total^4^.

### Metrics

#### Logical error per round

If *E*(*n*) denotes the decoder error rate (computed as the erroneous fraction of logical error predictions) at stabilizer measurement round *n*, we can make an ansatz for its functional dependence on *n* via

2 $$E(n)=\frac{1}{2}(1-{(1-2{\epsilon })}^{n}),$$

following previous work (supplementary material, equation (3) in ref. ^14^). For equation (2), we can see that *E*(0) = 0 (that is, we assume no error at round *n* = 0), *E*(1) = *ϵ* and *E*(*n*) approaches 1/2 for larger *n*. In this context, the quantity *ϵ* is called the LER; indeed, it describes the exponential decay of the decoder’s fidelity *F*(*n*)

3 $$F(n):= 1-2E(n)={(1-2{\epsilon })}^{n}.$$

How we obtain the LER *ϵ* from the error rates depends on whether we consider results after multiple different number of rounds, or whether we derive it from an experiment with a unique number of rounds (say, for instance, 25). The 2 ways of deriving the metric are compatible, in the sense that performing a fit on an experiment with a single number of rounds (with the added constraint of setting *F*(0) = 1 explicitly) yields exactly the same LER as inverting the error *E*(*n*) directly via equation (4).

##### Experiment at a fixed number of rounds

For an experiment at a fixed number of rounds (for example, *n* = 25) we simply invert equation (2), and obtain

4 $${\epsilon }=\frac{1}{2}(1-\sqrt[n]{1-2E(n)}).$$

##### Experiment across multiple rounds

We determine *ϵ* via a linear fit of the log fidelity

5 $$\log F(n)=\log {F}_{0}+n\log (1-2{\epsilon }).$$

**Extended Data Fig. 3:**
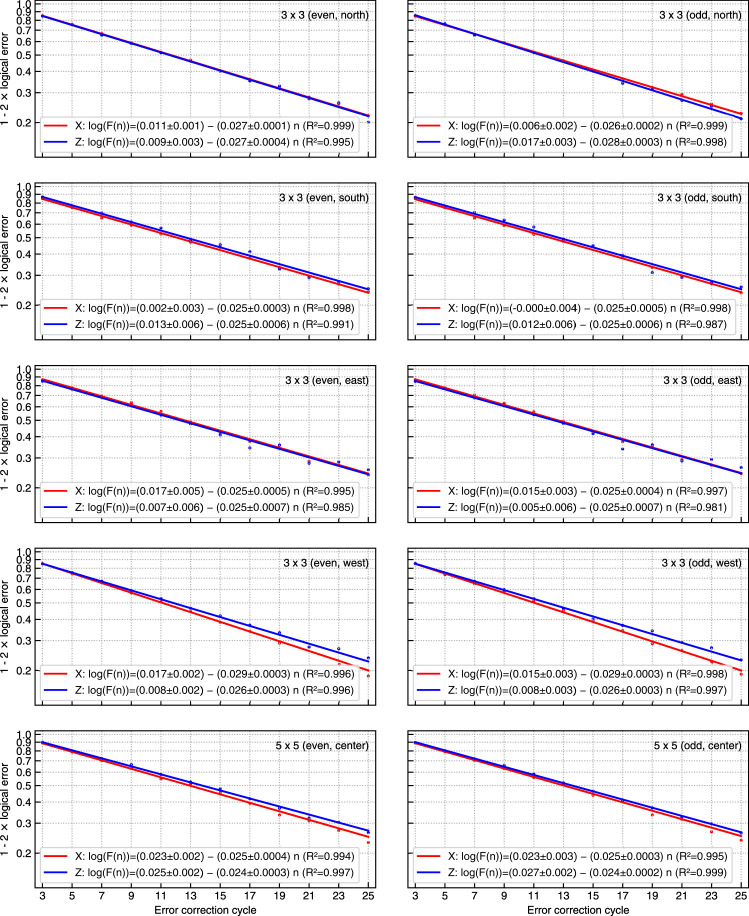
Individual fits of logical error per round for **3 × 3** and **5 × 5** memory experiments. For the *p*_*ij*_-DEM pretrained model.

To assess the fit’s quality, we use the goodness of fit, *R*^2^. In addition, we expect *F*_0_ to be close to *F*(0) = 1, so we consider significant departures of log*F*_0_ from 0 to indicate a bad fit (see ‘Termination’). As shown in Extended Data Fig. 3, all our fits for the 3 × 3 and 5 × 5 memory experiments show *R*^2^ ≥ 0.98 and *F*_0_ ≥ 1.

As done in the original Sycamore experiment^4^, we exclude the point (1, *E*(1)) from our fits due to a time boundary effect, which yields a much stronger error suppression per round at the first error-correction round.

### Note on statistics

#### Combining different datasets

In the experimental datasets (for example, Fig. 3b and Extended Data Fig. 9a), we have 16 (for code distance 3) or 4 (for distance 5) distinct datasets per aggregated model performance—the combination of *X* and *Z* bases, even and odd subsets, and the different device regions. As we do not expect performance on the different datasets to be the same, we purposefully exclude the spread across datasets from our error estimation. Our error estimation is derived exclusively from the bootstrap estimation (499 resamples) of the individual fidelity points. Consistent with the literature^4^, we propagate individual fitting errors by Gaussian error propagation; that is, we sum two quantities *e*_1_ ± d*e*_1_ and *e*_2_ ± d*e*_2_ via $e=({e}_{1}+{e}_{2})\pm \sqrt{{\rm{d}}{e}_{1}^{2}+{\rm{d}}{e}_{2}^{2}}$, discarding the spread between the quantities.

#### Combining different seeds

When we have multiple seeds but only one dataset (for example, the ablation for Pauli+ data, Extended Data Fig. 9b), we exclusively consider the spread across datasets, discarding the bootstrapped variance of the individual samples.

#### Many-rounds experiments

We use 9 bootstrap resamples.

### Model details

AlphaQubit is a neural network designed to decode the surface code for a range of code distances and for experiments of arbitrary duration. Here we describe the features of the architecture, particularly those that are adapted to the quantum error-correction problem. The ‘Ablations’ section shows that several of these become more important for the more complex decoding problem at larger code distances. Pseudocode for the model can be found in Supplementary Information.

**Extended Data Fig. 4:**
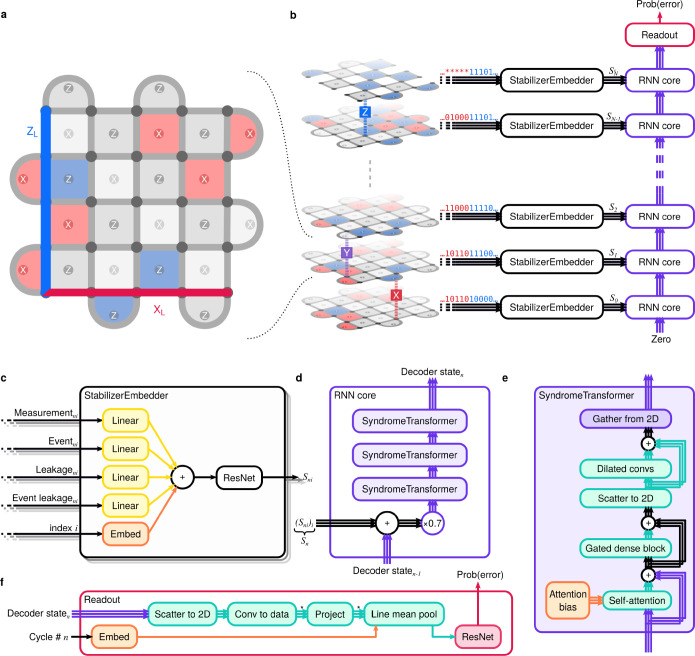
The neural network architecture designed for surface code decoding. (**a**) 5 × 5 rotated surface code layout, with data qubits (dark grey dots), *X* and *Z* stabilizer qubits (labelled light grey dots, or highlighted in blue/red when they detect a parity violation) interspersed in a checkerboard pattern. Logical observables *Z*_L_ and *X*_L_ are shown as bold lines on the left and bottom grid edges respectively. (**b**) The recurrent network iterates over time updating a representation of the decoder state and incorporating the new stabilizers at each round. Three parallel lines indicate a representation *per stabilizer*. (**c**) Creation of an embedding vector *S*_*ni*_ for each new stabilizer *i* = 1, …, *d*^2^ − 1 for a distance *d* surface code. (**d**) Each block of the recurrent network combines the decoder state and the stabilizers *S*_*n*_ = (*S*_*n*1_, …, *S*_*nM*_) for one round (scaled down by a factor of 0.7). The decoder state is updated through three Syndrome Transformer layers. (**e**) Each Syndrome Transformer layer updates the stabilizer representations through multi-headed attention optionally modulated by an (optional) learned attention bias followed by a dense block and dilated 2D convolutions. (**f**) Logical errors are predicted from the final decoder state. The triple lines marked with * indicate a representation *per data qubit*.

The recurrent architecture design (Extended Data Fig. 4b) reflects the time-equivariant nature of the problem with the syndrome transformer maintaining the decoder state, which represents the information from previous stabilizers relevant to deciding an experiment outcome. The decoder state has the potential to store some information for a window of arbitrary duration and is not limited to a fixed window.

#### Input representation

The network is provided with between one and four inputs per stabilizer. In the simplest case, analogous to MWPM, we provide binary detection events, which are the temporal differences of binary measurements of the stabilizer state. Although these contain the same information, in practice we find that measurement inputs lead to better results than event inputs alone, so we provide both (Extended Data Fig. 9). There are several possible factors at play here that might explain the usefulness of providing measurements: they have a more uniform distribution than events, making the model input less biased. Furthermore, for the Pauli+ data, providing measurements may help resolve the asymmetry of the states $| 1\rangle$ versus $| 0\rangle$ (due to the amplitude damping component in the noise model; see ‘Measurement noise’); yet after translation to events, the information whether the event was due to a flip $| 0\rangle$ to $| 1\rangle$ versus the other way round is lost. When simulating I/Q noise, we thus provide both measurements and events as probabilities as described in ‘Soft measurement inputs versus soft event inputs’. For experiments with leakage, we also provide the leakage probability and the temporal-difference analogue. Although the soft model input is presented at float32 values to the model, we anticipate that a much lower bit precision (such as eight or four bits) would suffice to capture most of the benefit of soft readouts.

A representation is built up for each input stabilizer as shown in Extended Data Fig. 4c, by summing linear projections of each of the input features. This means that there are *d*^2^ − 1 different embeddings generated in the ‘StabilizerEmbedder’, which are then added to the *d*^2^ − 1 representations that comprise the decoder state (Extended Data Fig. 4d). To allow the transformer to distinguish between the stabilizers, we also add a learned input embedding of the stabilizer index *i*. As the final-round stabilizers are not measured but computed from the data qubits, we encode this by using a separate embedding for the final round, with a separate final-round linear projection for the on-basis computed stabilizers and a single learned embedding for all the undefined off-basis stabilizers (where on- and off-basis stabilizers are defined in ‘The rotated surface code’). Each stabilizer representation is independently passed through a two-layer residual network to derive the representation, *S*_*ni*_, provided to the recurrent neural network (RNN) for stabilizer *i*. (${S}_{Ni}^{{\prime} }$ for the final stabilizers).

At each error-correction round, the stabilizer representations are added to the corresponding decoder state vectors and then scaled by a factor 0.707 to control the magnitude (Extended Data Fig. 4d). This code-distance-independent constant is intended to prevent the scale of the state vectors from growing, designed so if both inputs are zero-mean unit variance, the scaled-summed output will also be.

We emphasize that although the noise model used in the scaling experiment (Pauli+, which simulates effects such as cross-talk and leakage, and augmented soft readouts with amplitude damping) is more realistic than a circuit depolarizing noise model, we only provide AlphaQubit with these stabilizer measurements and events (plus leakage information) described above. There is no privileged access to information for cross-talk or other noise events from the simulators, beyond what one could measure in an actual memory experiment. AlphaQubit learns these noise effects beyond the SI1000 circuit depolarizing noise prior solely from finetuning.

Similarly, as explained in ‘Detector error model’, the DEMs used for the matching-based decoders are all derived from a circuit depolarizing noise prior, and then either fitted to the Sycamore experimental data, or the noise parameters manually adjusted to match the noise effects within the Pauli+ and I/Q noise simulations. As such, the DEMs do not directly capture any of the cross-talk effects present in the experimental data or simulation (beyond what can be captured in the fit and parameter adjustment).

#### Syndrome transformer

We designed the computation block (Extended Data Fig. 4d) to match the quantum error-correction task. At the heart of our RNN block architecture is the syndrome transformer, a self-attention architecture based on the transformer^66^, which has seen recent success in a variety of problems^67,68^. Transformers consist of multi-head self-attention followed by a fully connected dense block that we augment with gating^69^, which modulates the dense block’s computation with multiplicative factors between zero and one. To this, we add two elements: two-dimensional convolutions, to promote better scaling with code distance and inspired by the space-translation symmetry of the problem, and (optionally) attention bias, which precomputes a component of the attention and adds a degree of interpretability (Extended Data Fig. 4e).

The syndrome transformer updates the per-stabilizer decoder state representation by incorporating information from other stabilizers based on their location. Although previous decoders have exploited symmetries of the toric code^70^ and used convolutional neural networks for processing the surface code^71^, boundary conditions of the surface code together with non-uniformity of real physical devices mean that there could be advantages to a model that goes beyond rigid spatial invariance. Much of the information passing can be local, to handle local spatial correlations, and can be modelled with two-dimensional convolutions. Longer-range interactions depending on relative stabilizer location are partially supported by dilated convolutions (in which a 3 × 3 convolutional kernel is ‘spaced out’ to skip intervening pixels and model longer-range dependencies). Dense all-to-all attention enables the model to dynamically reason about all possible stabilizer pairs depending on their current state. Such a pairwise attention mechanism is useful in capturing (possibly long-range) correlations during decoding, much like MWPM finds edges in the decoder graph between pairs of detection events.

For each transformer layer, we apply three dilated convolutions after first scattering the stabilizer representations to their two-dimensional spatial layout in a (*d* + 1) × (*d* + 1) grid, with an optional learned padding vector for locations where there is no stabilizer.

#### Attention bias

The attention allows information exchange between all pairs of stabilizers. We expect that this attention between any two stabilizers is dependent on two factors: the history of events for each of those stabilizers and the relationship of those stabilizers in the circuit (in terms of basis, connectivity and spatial offset). As the relationship between the stabilizers is constant, we learn an attention bias that modulates the attention between stabilizer *i* and *j*. The attention bias is a precomputed offset to the attention logits, learned separately for each head in each transformer layer.

The attention bias embeds fixed information about the layout and connectivity of the stabilizers by constructing a learned embedding for each stabilizer pair *i*, *j* as a function of *i* and *j*. This embedding is independent of the decoder state and at each transformer layer is projected down to a scalar bias per head to be added to the conventional content-based attention logits.

The attention bias embedding is a (*d*^2^ − 1) × (*d*^2^ − 1) × 48 tensor constructed by adding learned embeddings of discrete features for each stabilizer pair *i*, *j* based on their spatial layout. The features are chosen to encapsulate salient attributes of the spatial relationship between stabilizers.

1. The spatial coordinates of stabilizer *i* and stabilizer *j*.
2. The signed spatial offset of stabilizer *i* from stabilizer *j*.
3. The Manhattan distance between *i* and *j*.
4. A bit to indicate if the stabilizer types (by which we mean the basis labels inherited by transforming the *X*-type and *Z*-type stabilizers from the usual CSS code, as explained in ‘The rotated surface code’) for *i* and *j* are the same or not.

These learned embeddings are then independently passed through a residual network to form the final embedding. Although the embedding is learned, after training the attention bias is constant and can be precomputed.

To provide a simple further modulation of the bias, at each round, the current and previous stabilizers are used to compute indicator features for spatial and time–space event correlations^4^ for each *i*, *j* pair. At round *n* these are the products:

1. event_*ni*_ × event_*nj*_ (spatial)
2. event_*ni*_ × event_(*n*−1)*j*_ (time–space)
3. event_(*n*−1)*i*_ × event_*nj*_ (time–space)
4. event_(*n*−1)*i*_ × event_(*n*−1)*j*_ (spatial)

as well as the diagonals of these (seven features as two diagonals are identical).

These features are concatenated to the attention bias embedding and directly projected to the attention bias with a learned projection. Although, for speed reasons, only simple binary features are provided and projected directly to the bias, with the additional features only the attention bias embedding can be precomputed and the projection becomes costly for large code distances. The ablations show that the attention bias adds little to the performance, and it is more costly during training, so although the ablations of Extended Data Fig. 9a,b are relative to a baseline with attention bias, the Pauli+ experiments were executed without it.

##### Attention bias visualizations

**Extended Data Fig. 5:**
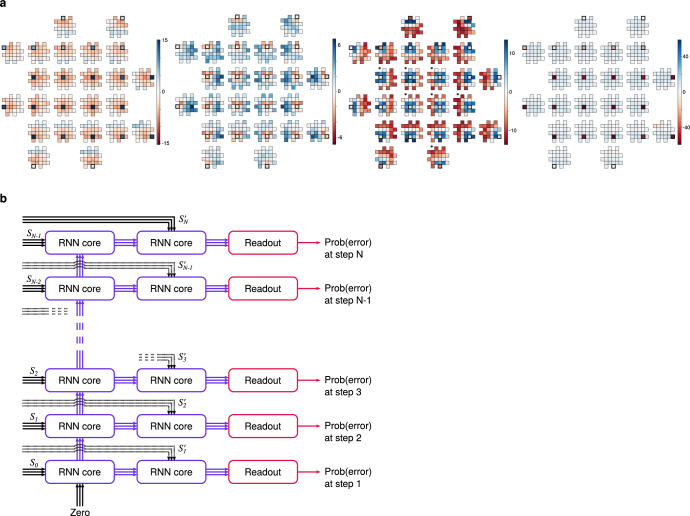
Further architecture details. (**a**) Attention bias visualisation. Attention bias logits of the four heads of the first Syndrome Transformer layer of our decoder model pretrained on 5 × 5 DEM in the Z basis. We obtain the logits by combining the learned attention bias embedding with all-zero stabilizer values. The 24 × 24 attention logit matrices are each visualized as one grid per stabilizer, laid out according to the physical layout of the attending stabilizer qubits. Each grid shows the logits for the attention to each stabilizer, with self-attention highlighted with a black square. (**b**) Architecture of the network when predicting labels at every round. *S*_*n*_ are the stabilizer representations as explained in Extended data Fig. 4b, where the primed quantities $S{{\prime} }_{n}$ indicate the embeddings are computed using different embedding parameters and based only on the stabilizers in the experiment basis computed from the final data qubit measurements (‘Input representation’ in Methods).

To investigate whether the attention bias learns an interpretable representation, we visualize its logits in Extended Data Fig. 5. For each of the 4 attention heads for the first transformer layer of one (5 × 5) DEM-trained model, we plot the attention logits for each stabilizer in a physical layout. It clearly shows that the different attention bias heads perform distinct functions. The first head modulates the attention towards the same stabilizer and stabilizers far away in the surface code. The second head discourages attention to immediate neighbouring stabilizers (even more so to the diagonally adjacent stabilizers between on- and on-, respectively off- and off-basis stabilizers; see ‘The rotated surface code’) while encouraging attention to non-neighbouring stabilizers. The third head instead does the opposite, strongly encouraging local attention while discouraging attention to stabilizers farther away. In addition, the third head seems to show patterns of higher attention biases for on-basis (see ‘The rotated surface code’) stabilizers than for off-basis stabilizers. This is visible in the attention maps marked with an asterisk. Lastly, the final head predominantly discourages attention to the same stabilizer while being slightly encouraging towards attention for non-same stabilizers. We observed similar patterns of local and non-local attention bias for other models; however, it did not always show as clearly and in some models the attention bias offered no obvious interpretation.

#### Readout network

After the RNN has processed the final stabilizers from round *N* to create the decoder state_*N*_ representation, a readout network (Extended Data Fig. 4f) processes the state to make a final prediction, again using the spatial distribution of the stabilizers. In the readout network, we first transform the per-stabilizer representation to a per-data-qubit representation by a scatter operation, which arranges the decoder state representation according to the stabilizers’ spatial layout and then applies a 2 × 2 convolution that combines information from the 4 stabilizer neighbours of each data qubit. We then apply a dimensionality reduction and mean pooling along rows or columns of the data qubits perpendicular to the logical observable rows or columns of qubits (depending on the measurement basis), to arrive at a vector representation per equivalent logical observable. This representation is then processed by a residual network to make the final label prediction. We can compute one label for each of the *d* rows or columns corresponding to equivalent choices of logical observables in the experiment basis (Extended Data Fig. 4a) and average the loss for all of these if all the labels are available (as they are for the scaling experiment simulations). Only a single logical observable is used at inference time (the leftmost or the lowest according to the basis). The network was designed to pool along logical observables to give a prediction per line, but in practice we found better results pooling perpendicular to them.

#### Auxiliary tasks

Often, training neural networks to make predictions other than those required for the main machine-learning task, known as auxiliary tasks, can lead to improved training or better performance on that main task. Here we ask the network to make a prediction of the next stabilizers, by a linear projection and logistic output unit from each stabilizer’s representation. Extended Data Fig. 9 shows that this auxiliary task seems to detract slightly from the network performance, but leads to slightly faster training.

#### Efficient training for variable-duration experiments

As the computation could be terminated at any round, the network could be asked to make a prediction at any round. We use our privileged access to the quantum state during simulation to provide a label for any round (see ‘Intermediate data’). It is noted that owing to the special nature of the final stabilizers being computed from the measured data qubits, there is a set of final stabilizers for round *N* that are different from the bulk stabilizers for round *N* of experiments that last longer. With such simulated data, when training, we can share computation for these experiments of different lengths as shown in Extended Data Fig. 5b. For *N* rounds, *N* labels can be trained with 2*N* applications of the embedding and RNN core (*N* for the bulk and *N* for the final stabilizers for each duration), and *N* readout computations. (versus *N*(*N* + 1)/2 applications of the embedding and RNN with *N* readout computations for training on *N* separate examples of durations 1, …, *N*).

### Implementation details

Our machine-learning decoder architecture is implemented and trained using the JAX, Haiku and JAXline^72^ machine-learning frameworks.

### Training details

#### Sycamore data

##### Cross-validation

For the data from Google’s Sycamore memory experiment paper^4^, we consider the two disjoint sets of odd- and even-indexed experiments (see ‘Sycamore memory experiment dataset’) to perform twofold cross-validation.

##### Pretraining

Owing to the limited amount of experimental data captured in the Sycamore experiment, compared with the number of training examples required for a machine-learning decoder, we pretrain on simulated data with three different degrees of similarity to the experimental data. The first one is data generated from a previously published DEM file^4^, obtained by fitting one half of the experimental data (either odd- or even-indexed, 25,000 samples per experiment length, see ‘Detector error model’). Examples are uniformly sampled from the lengths {1, 3, …, 25}. We use simulated samples from the DEM fitted to the same half as validation dataset; which we use to perform early stopping, that is, to select the model parameters producing the minimum LER on the validation dataset across training steps. We stop the experiment before using 2 billion training samples.

When XEB pretraining, we follow a similar procedure, but using data generated from a DEM file derived from a previously published Stim file^4^, obtained using device calibration data, for both training and evaluating.

In the last pretraining variety, SI1000 pretraining, we train and evaluate with SI1000 data not fitted to the device (see ‘Circuit depolarizing noise’). Instead of simulating experiments of lengths {1, 3, …, 25}, in the SI1000 pretraining modality, we always simulate experiments of length 25 and use intermediate measurements as auxiliary labels at each training rounds (see ‘Intermediate data’).

For all three varieties of pretraining, we use a ‘noise curriculum’ in which we show data with lower noise strength at the beginning of the training, continuously transitioning to higher noise strength during the training process. More precisely, for DEM and XEB pretraining, we consider several replications of the DEM with error event probabilities scaled with factors *f* = 0.5, 0.6, …, 1.0; whereas for SI1000 pretraining, we scale the parameter *p* accordingly. Each dataset is sampled with a probability proportional to

6 $${p}_{{\rm{f}}}(t)\propto 1+{w}_{{\rm{c}}}G(\,{f}_{{\rm{c}}}(t),{\sigma }_{{\rm{c}}},f)$$

where *G*(*μ*, *σ*; *x*) is the standard un-normalized Gaussian function and *f*_c_(*t*) is the peak scale factor:

7 $${f}_{{\rm{c}}}(t)={f}_{{\rm{c,min}}}+\frac{1-{f}_{{\rm{c,min}}}}{1+\exp (-{s}_{{\rm{c}}}(t/{t}_{{\rm{c}}}-1))}$$

that transitions from the minimum peak scale factor, *f*_c,min_ to 1 at a number of training steps *t* = *t*_c_. The values of the noise curriculum parameters can be found in Extended Data Fig. 8a. We found that this noise curriculum stabilizes training, and yielded slightly better accuracy at the end.

##### Finetuning

We then finetune the model using one half of the experimental data (for the DEM pretrained case, always the half used to derive the DEM file). We further divide this half of the data, and use the first 19,880 samples as a training dataset and the remaining 5,120 samples as a development dataset for early stopping, keeping the parameters giving the best fitted LER (up to 30,000 training steps). The final model is evaluated on the other half of the experimental data—the 25,000 held-out samples not used for training or early stopping. The best validation LER is found after about 120 passes through the data.

#### Pauli+

##### Pretraining

We train and evaluate the model on SI1000 data generated using Stim with added I/Q noise (see ‘Measurement noise’), with intermediate measurements as auxiliary labels (see ‘Intermediate data’). The I/Q noise is simplified in that we set *t* = 0 (that is, no amplitude damping component). Furthermore, before each measurement in the simulated experiment, we first randomly set the qubit to a leaked $| 2\rangle$ state with a 0.275% chance, and then add I/Q noise. This is the only source of leakage that we add to the system; Stim itself (unlike Pauli+) does not simulate leakage (or cross-talk).

Although the amount of leakage is approximately matched to the Pauli+ data, this is a very simplified simulation of leakage in the system compared with ‘Pauli+ model for simulations with leakage’. Here we treat leakage as an effect that just occurs during measurements; whereas in the Pauli+ simulation leakage occurs through realistically modelled effects during the application of quantum gates, and also spreads accordingly. One consequence is that in the Pauli+ data, the amount of leakage between, for example, stabilizer and data qubits varies strongly, an effect we disregard during pretraining. Nonetheless, providing the model with this very simplified version of leakage (through the same inputs post_1_ and post_2_ described in ‘Measurement noise’) helps prime our decoder to expect leakage information as input. Examples all have 25 error-correction rounds.

##### Finetuning

For the finetuning, we trained models using samples from the Pauli+ simulator (see ‘Pauli+ model for simulations with leakage’) using 15 different seeds. We trained with either soft or hard inputs, with approximately 0.1% chance of leakage in the stabilizer measurements and with I/Q hyperparameters: SNR = 10 and *t* = 0.01. We used the auxiliary task of predicting the next stabilizers but not intermediate labels as these are not available in a realistic scenario. After termination, the model parameters obtaining the lowest development set cross-entropy loss were chosen.

For each code distance 3, 5, 7, 9 and 11, we generated 100 million training samples and 5 million test samples from the Pauli+ simulator, which were then augmented with I/Q noise in post-processing. For each data limit from Fig. 4, we sampled a subset from the training samples and split off about 10% as a development set. The models were trained for up to 10 epochs.

#### Loss

We trained the model using cross-entropy objectives with binary targets. For the scaling experiments, where we have a label for each logical observable for each experiment duration, all these losses were averaged.

As an auxiliary loss, we used next stabilizer prediction cross-entropy loss (see ‘Auxiliary tasks’) averaged across all rounds and all stabilizers and then down-weighted relative to the error-prediction loss (Extended Data Fig. 8b).

#### Loss minimization

We minimize loss using stochastic gradient descent. We use the Lamb^73^ and Lion^74^ optimizers for experimental and scaling datasets, respectively. We use weight decay (L2 norm on non-bias parameters) everywhere, either relative to zero (for pretraining) or relative to pretrained parameters (for finetuning, using a stronger weight decay). The batch size was increased once (from 256 to 1,024) during pretraining (Extended Data Fig. 8b) and kept at 1,024 for finetuning.

The learning rate is piecewise constant after an initial linear warm-up of 10,000 steps, with reductions by a factor 0.7 at specified numbers of steps. Scaling experiments used a cosine learning-rate schedule. For the Pauli+ experiments, we pretrain with a rounds curriculum; for example, for a 25-rounds experiment, we train for 30 million seen examples on 3 rounds, from 30 million to 60 million examples on 6 rounds, from 60 million to 90 million examples on 12 rounds, and above that on 25 rounds.

#### Termination

Pretraining is terminated after 2 billion examples for the Sycamore experiments with *p*_*ij*_ and XEB and 500 million examples for SI1000. For the scaling runs, pretraining was terminated after up to 2.5 billion examples for the scaling runs. Model parameters are accumulated with an exponential moving average and regularly evaluated on development data to compute the LER (by fitting across rounds 3, 5, …, 25 for the Sycamore data, or by computing for 25 rounds for the scaling data). The set of parameters with the lowest development set LER is retained. With noisy fidelity estimates, particularly early on in training, we found that LER could be overestimated (see ‘Logical error per round’), so we exclude parameter sets for which the fit has *R*^2^ ≤ 0.9 or an intercept ≤max(−0.02, −*σ*) where *σ* is the estimated standard deviation for the intercept of the fit line. For the scaling finetuning experiments, as the constrained development data would in some cases be too small to reliably estimate the low LERs, the lowest development set cross-entropy was used to select the model parameters.

#### Hyperparameters

For the Sycamore experiments, we tuned the hyperparameters of the network by training models on the 5 × 5 DEM, using samples from the same DEM as validation data for hyperparameter selection. We used the same hyperparameters for 3 × 3 except for learning rate ($\times \sqrt{2}$) and using dilation 1 convolutions (see Extended Data Fig. 8c for details).

For the scaling investigation, the same base model was used, with some hyperparameter values further tuned to minimize validation set LER for the 11 × 11 code. Again, the same model is used for all other code distances except for adjusting the learning rate and choosing the convolution dilations (see Extended Data Fig. 8c for details).

#### Parameters

As the architecture used for all code distances is the same, the number of parameters (the weights of the neural network) is constant except for additional stabilizer index embedding parameters needed for larger code distances. All the convolutions are 3 × 3, albeit that the dilations are varied with the code distance.

### Ensembling

It is possible to combine multiple classifiers to obtain a more accurate prediction by ensembling^27,75^. As the models can be run independently in parallel, ensembling does not change the computation speed, but does require more computational resources. We apply ensembles of multiple models for both the Sycamore and scaling experiments. Extended Data Fig. 7d shows the improvement in LER owing to ensembling compared with the average performance of the models used in the ensemble.

We train multiple models with identical hyperparameters, but different random seeds leading to different parameter initializations and training on different sequences of examples. We use 20 seeds for the Sycamore data and 15 for the scaling experiments. We average the logits from the different networks (computing a geometric mean of the predicted error probabilities). As there is a range of accuracy between seeds, it might be possible to achieve greater performance by ensembling only the models with the best validation set performance, but here we ensemble all the models trained.

### Decoding speed

We designed and tuned the current model to deliver the best error suppression possible up to 11 × 11 codes, with the only speed consideration being to keep training time manageable for experiments. So far, we have put no effort into optimizing the design for inference speed. When running AlphaQubit on an accelerator for code distances up to 25 (but untrained for distances >11), its throughput is within 1–2 orders of magnitude above the target throughput rate of 1 μs (ref. ^20^), and within an order of magnitude of the throughput of, for example, PyMatching^76^, an implementation of standard MWPM without correlations, analogue inputs or calibrated error probabilities (Extended Data Fig. 7a).

Throughput measures only the time required for computation per round, ignoring the latency—the time required to deliver a final answer after receiving the final round’s stabilizers.

By design, the model runtime is independent of the physical noise level (and hence the error syndrome densities), whereas matching is slower the greater the noise. The fixed runtime of neural network decoders is considered to be a practical advantage^77^.

We are confident that our decoder can be sped up significantly using a number of well-known techniques. Although well studied, the optimization of neural networks for speed in deployment is a multistep process to be undertaken after demonstrating the accuracy of our approach at the target scale. First, having established the accuracy that is achievable, optimizing the architecture and hyperparameters for inference speed is likely to deliver significant gains while maintaining that accuracy. For instance, improving parallelism and pipelining are expected to improve throughput, perhaps at the expense of incurring greater latency. Using faster components such as local, sparse or axial attention^78^, which restrict attention to a subset of stabilizer pairs, have the potential to deliver speed improvements. Custom implementation to optimize for a particular hardware accelerator design and to remove memory access bottlenecks can also improve speed for a specific architecture.

Second, techniques such as knowledge distillation^79^ and attention transfer^80^, lower-precision computation, and weight and activation pruning can be applied to achieve similar performance with less computation. In knowledge distillation, we first train a large, accurate ‘teacher’ network on the task. Then we train a smaller ‘student’ network whose architecture is tuned for inference speed^81^. In addition to the logical observable labels that were available to the teacher, the student network is trained to match the probabilistic outputs of the teacher. Optionally, the student can be trained to match the internal activations and attention maps of the teacher. These richer, denser targets have been shown to enable the student to achieve higher accuracy than could be achieved when training the student network without the teacher network. For increased accuracy, an ensemble of teachers can be distilled into a single student.

We note that machine-learning acceleration hardware is continually improving (for example, one study^82^ found a factor of 2 increase in floating point operations per second about every 2.6 years). Should this trend continue and we can choose architectures to exploit the improvements, this would lead to considerable speed-up over the timescale anticipated for the development of full-scale quantum computers.

Finally, custom hardware-specific implementation on application-specific integrated circuits or field-programmable gate arrays can deliver further speed improvements. Previous studies have demonstrated the feasibility of implementing decoders (for example, Union Find^54^ and neural network^83^) in such custom hardware. To best exploit application-specific integrated circuits and field-programmable gate arrays, low precision, fixed-point arithmetic and sparse networks may be necessary.

We also note that, in principle, it is possible to achieve unbounded throughput by decomposing the matching decoding problem in an extremely parallel fashion^55,56^. We expect similar ideas might be applied to a machine-learning decoder (‘Generalization to logical computations’ below).

### Generalization to logical computations

Beyond scaling a decoder to achieve sufficiently low logical error rates (see ‘Further considerations of scaling experiments’) and ensuring throughput rates commensurate with hardware demands (see ‘Decoding speed’), realizing a fault-tolerant quantum computation requires a decoder to handle more than memory experiments, such as lattice surgery operations. Despite significant recent progress on the experimental side^4,17–21,84–87^ and decoding side^25–27,88–90^, decoding a logical computation is only now beginning to be explored, even for established decoding schemes such as MWPM^58^.

One possible approach (akin, on a high level, to the spatial and temporal windowing approach presented for graph-based decoders^55,56,58^) is to train separate network components to implement different building blocks of a fault-tolerant quantum circuit. These could be trained individually (as demonstrated for idling in the memory experiment) and across constructed combinations, to ensure that the state information passed between them is consistent and sufficient for decoding arbitrary circuits. We expect that many, perhaps all, of the network parameters could be shared, such that a single model generalizes across different decoding scenarios, perhaps utilizing side inputs to indicate the gate required, and producing auxiliary outputs that allow decoders on different building blocks to exchange information necessary for the joint decoding task.

**Extended Data Fig. 6:**
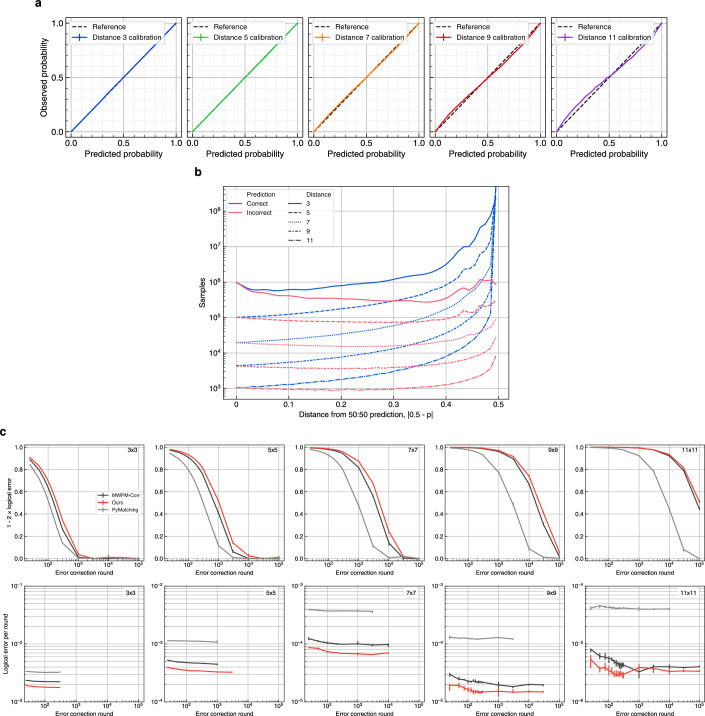
Calibration of AlphaQubit’s outputs and generalization to longer experiments for code-distances 3–11. (**a**) Calibration for Pauli+ generated data with SNR = 10, *t* = 0.01, and 0.1% stabilizer qubit leakage chance. (**b**) Calibration histogram of predicted probabilities. The predictions are grouped into correct (blue) and incorrect (red) before building the histogram, and then binned into “certainty” bins depending on their distance from a 50: 50 prediction, i.e. by ∣1/2 − *p*∣ for a predicted probability *p*. For all code distances, wrong predictions have a lower certainty. Correct predictions concentrate around the interval edges, i.e. at probabilities 0 and 1, resulting in a high certainty. (**c**) Generalization to longer experiments. 1 - 2 × logical error and logical error rates for networks pretrained and finetuned on datasets for up to 25 error detection rounds but applied to decoding experiments of longer durations. We only plot logical error rates where the corresponding 1 - 2 × logical error are greater than 0.1. The data is generated from the same simulated experiments, stopped at different number of rounds.

**Extended Data Fig. 7:**
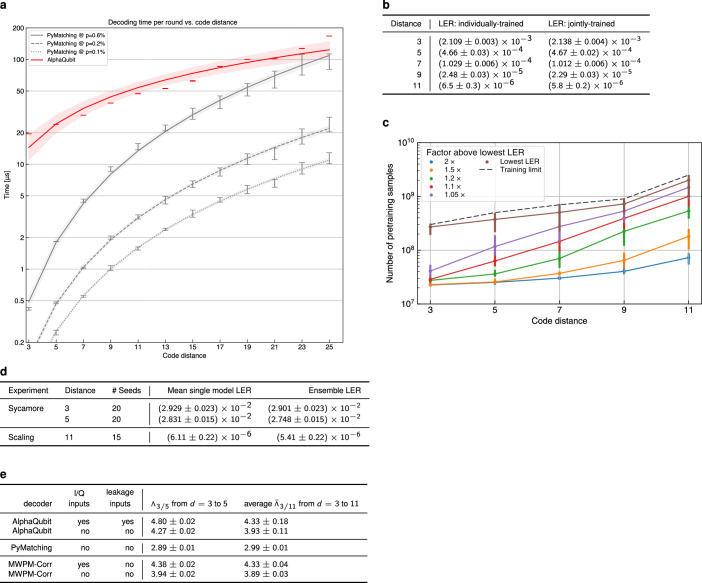
Further result details on Sycamore and scaling experiments, and decoder speed. (**a**) Decoding time per error correction round vs. code distance. The hatched region for *d* > 11 indicates that while AlphaQubit is the same for all code-distances, it has not been trained or shown to work beyond *d* = 11. The line is a least squares fit to *a* × *d*^exponent^, and the shaded region marks a 95% CI. Timing of uncorrelated matching (PyMatching). Times for PyMatching use a current CPU (Intel Xeon Platinum 8173M) and for AlphaQubit use current TPU hardware with batch size 1. (**b**) LER of a decoder trained jointly on distances 3–11, as compared to decoders trained solely on the individual code distances (both pretrained only, see ‘Decoding at higher distances’ and ‘Pauli+’ in Methods). Uncertainty is bootstrap standard error (9 resamples). The model and training hyperparameters are identical in both cases, but for the joint code distance-trained decoder we swap the index embedding for a relative positional embedding, where each stabilizer position (normalized to [−1, 1] × [−1, 1]) is embedded (‘Input representation’ in Methods). (**c**) Number of pretraining samples until the individual models (pre ensembling) achieve the given LER (relative to the lowest-achieved LER, the latter shown as brown line). The dashed line indicates when the training was stopped (see ‘Termination’ in Methods). Error bars are 95% CI (N = 15). (**d**) Performance improvement by ensembling multiple models, where we show the *p*_*ij*_-pretrained model for the Sycamore experiments (XEB and SI1000 variants show about the same improvement). (**e**) Average error suppression factors **Λ** for Pauli+ experiment. The error suppression factor is computed from the data in Fig. 4, via the geometric average ${\bar{\Lambda }}_{3/11}={({{\epsilon }}_{3}/{{\epsilon }}_{11})}^{1/4}$, for a logical error per round *ϵ*_3_ at code distance 3, and *ϵ*_11_ at distance 11, respectively.

The ability of our neural architecture to transfer between various decoding scenarios will be crucial in this context. Beyond generalization across rounds, that is, the streamability of the decoder (Fig. 5 and Extended Data Fig. 6c), we can also successfully train a single machine-learning decoder across multiple code distances. More specifically, in the ‘Quantum error correction on current quantum devices’ and ‘Decoding at higher distances’ sections, we present decoders that have been pretrained on the code distance on which the decoder will then be finetuned. This approach allowed us to assess each code distance independently, during both pretraining and finetuning. But the decoder can in fact be trained on a mixture of examples from a range of code distances, such that a single decoder can process any of the code distances it was trained on (Extended Data Fig. 7b). The resulting logical error rates of the cross-code-distance decoder are consistent with the model trained per code distance, when training up to the same number of samples seen in the single code distance *d* = 11 case. This form of generalization is enabled through the composition of architecture components (convolutions and attention) that can be applied independent of code distance.

### Further considerations of scaling experiments

In Extended Data Fig. 7e, we collect error-suppression factors *Λ* from distances 3 to 5 and distances 3 to 11, for various decoders and input modalities. The strongest error-suppression up to distance 11 is achievable with AlphaQubit, at an LER of (6.11 ± 0.22) × 10^−6^ (Extended Data Fig. 7d).

With our two-stage training procedure, the bulk of the computational training cost comes from pretraining the model to high accuracy. One model, pretrained on a generic noise model such as SI1000, can be copied and finetuned independently to various device characteristics, which amortizes the cost of pretraining. As detailed in the main text, these finetuning samples are a scarce experimental resource. In this work, we have shown that, up to code distance 11, we can finetune a model on a realistic number of experimental samples (10^7^; Fig. 4c). It is an important question for future research to demonstrate that this methodology remains successful at larger scales.

As we go from distance 3 to 11, the number of pretraining samples required for convergence increases, which, for example, manifests in Fig. 4, where the resulting relative advantage over MWPM-Corr is not as large at *d* = 11 as it is at *d* = 9. In our experience, the number of samples required to train a decoder at distance *d* seems to depend in a nonlinear fashion on the distance (Extended Data Fig. 7c). Extrapolating this trend is difficult, however, as we find that the sample growth rate strongly depends on the choice of hyperparameters, such as learning rate, batch size and architecture features. It could further be the case that finetuning a model at larger code distances to a fixed relative advantage over pretrained performance is more challenging than at smaller code distances: in Fig. 4c, we do not observe a significant improvement of the LER at distance 11 for 10^5^– 10^6^ finetuning samples compared with the pretrained model.

A possible partial explanation could be that the decreasing number of failure cases at larger distances (for example, only 0.025% for a 25-round experiment at an LER of 10^−5^) could result in fewer ‘challenging’ examples for learning, as seen in our distance-11 scaling demonstration where most samples are correctly classified late in training. The effectiveness of finetuning might be limited, similar to pretraining, because only a small portion of the data actually helps improve accuracy.

There are several possible approaches to improve sample efficiency. We have shown it is possible to train AlphaQubit across code distances instead of for a single code distance (see ‘Generalization to logical computations’). As we reach equal performance with the same total number of training steps as required to train a decoder for the highest code distance only, and as training steps for lower code distance examples are faster, training across code distances has the potential to save computational resources. Another approach that might improve sample efficiency is the concept of ‘hard sample mining’^91,92^, where difficult examples are collected (for example, by finding examples whose predictions are incorrect, or not confidently correct, for some decoder) or constructed. Training samples can then be biased towards these ‘hard samples’ rather than just drawing random samples. One indication that this is a fruitful avenue to pursue is that we have found benefit by training with noise levels higher than the intended target noise level, consistent with previous findings^36^. This could also work for finetuning, for example, in the form of injecting errors into the device to artificially collect high-utility data. Similarly, different finetuning mechanisms such as LoRA^93^ or few-shot learning strategies such as meta learning^94^ might prove fruitful.

Despite these arguments, training a machine-learning decoder at larger code distances will remain challenging. Correlated matching (and other, more recent graph-based decoders) will remain strong contenders going forward. Demonstrating that AlphaQubit can scale to distance 25 while maintaining competitive accuracy against MWPM-Corr will be one of the necessary steps (together with other decoding scenarios; see ‘Generalization to logical computations’) towards decoding a fault-tolerant computation at scale. We expect that improvements during training, as outlined above, and further hyperparameter adjustments and architectural improvements will be crucial to go to distances beyond 11 in an efficient manner.

### Time scalability

Ultimately, to perform fault-tolerant quantum computation at arbitrary circuit depths, a decoder needs to be able to maintain its decoding performance for an arbitrary number of error-correction rounds. Extended Data Fig. 6c shows the performance of networks trained up to 25 rounds and demonstrates that they maintain their performance when applied to much longer experiments, up to 100,000 rounds or until the 1 − 2 × logical error drops below 0.1.

We note that there appears to be a systematic decrease in LER with experiment length for the matching decoder, as well as AlphaQubit on smaller distances. We suggest that this is caused by the relatively high measurement noise in our simulations (inspired by a superconducting noise profile). This measurement noise is particularly damaging in the terminal round, where data-qubit measurements can cause space-like separated detection event pairs, which tend to be more damaging than time-like separated detection event pairs. This is further exacerbated by withholding I/Q information about these measurements from the machine-learning and MWPM-Corr decoders (see ‘Pitfalls for training on soft information’). Although we have chosen to train on 25 rounds to mirror previous work^4^, it would be interesting to quantify the effect of extending the training to more rounds, or finetuning for longer experiments.

We also note that although the recurrent architecture of our decoder means it uses a fixed amount of memory regardless of the experiment duration, PyMatching takes the entire matching graph as input, leading to memory consumption growing linearly with the experiment duration. We leave comparisons to streaming decoder implementations to future work.

### Soft matching

The MWPM decoder can also be augmented to use soft information^40^. For each I/Q point, we can compute the posterior probability that the sample was drawn from either the $| 0\rangle$-outcome distribution or $| 1\rangle$-outcome distribution—see ‘Measurement noise’. It is noted that this can sometimes classify $| 2\rangle$ outcomes as highly confident $| 1\rangle$ outcomes.

We can threshold these posterior probabilities to obtain binary measurement outcomes that are used to compute detection events. Then, the probability of the opposite outcome can be interpreted as a measurement error, which contributes a probability to one of the error edges in the error graph that instantiates the MWPM decoder. The probabilities of these edges are reassigned, replacing the average measurement probability contribution with these instance-specific probabilities. This change can be further propagated to the correlation reweighting step^25^. The posterior probabilities for data-qubit measurements are withheld to compare fairly with AlphaQubit, from which these values are also withheld. We leave comparison with a leakage-aware matching decoder, which reweights edges based on leakage detections^95^, to future work.

### Ablations

To understand the effect of different components of the architecture, we conduct ablations, by training networks where we remove or simplify one aspect of the main design and seeing the effect. For each scenario (described in the following sections), we trained 5 models with different random seeds and compare the mean test set LER in Extended Data Fig. 9a,b for 5 × 5 Sycamore DEM pretraining and 11 × 11 Pauli+ training respectively. For the former, Extended Data Fig. 9c also shows the effect on training speed. In each case, other hyperparameters were not changed, and it is possible that lost performance could be recovered by compensating with other changes (see ‘Decoding speed’). For ablations, we assess only pretraining performance.

Although many of the ablations have only a small effect on the performance at 5 × 5, at 11 × 11 the effects are more marked.

#### Model ablations

LSTM. We substitute the whole recurrent core with a stack of 6 LSTMs, as implemented in Haiku^96^. To keep the number of parameters roughly constant upon scaling (as AlphaQubit does), we make the width of the LSTM hidden layers dependent on the code distance *d*, and equal to 64 × (25 − 1)/(*d*^2^ − 1). As the LSTM uses dense layers, which lack any spatial equivariance, we also remove the scatter and mean pooling operations in the readout.

NoConv. We remove all the convolutional elements in the syndrome transformer.

**Extended Data Fig. 8:**
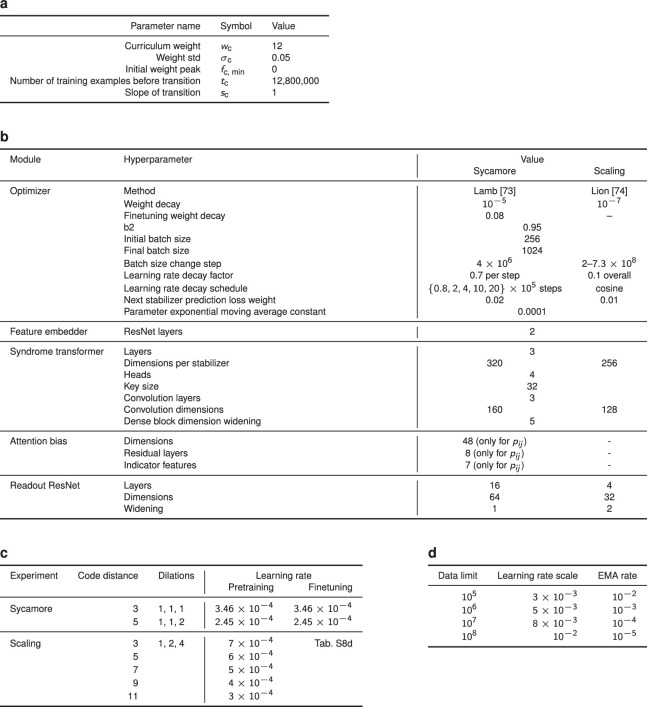
Network and training hyperparameters. (**a**) Noise curriculum parameters used in pretraining for the Sycamore experiment. (**b**) Hyperparameters of the network architecture. (**c**) The dilations of the three 3 × 3 convolutions in each syndrome transformer layer and the experiment learning rates are determined by the code-distance of the experiment. (**d**) Hyperparameters for finetuning of the scaling experiment. The learning rate for finetuning was the initial learning rate for the code-distance from Extended data Fig. 8c scaled by the factor dependent on the training set size. The finetuning cosine learning rate schedule length was code distance × 2/3 × 10^8^ samples.

**Extended Data Fig. 9:**
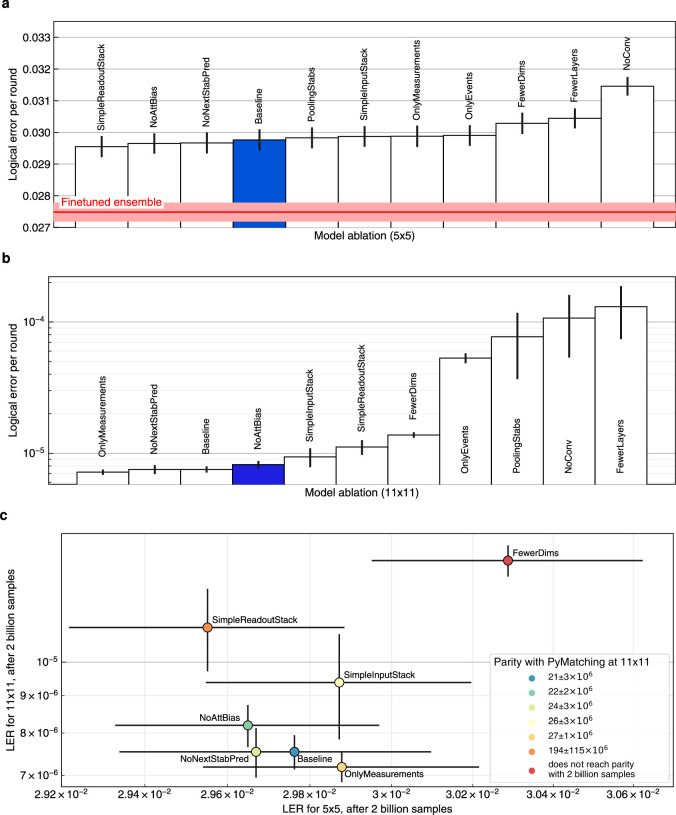
Ablations: The effect of removing or simplifying decoder architecture elements. Decoder performance under ablations (white bars) compared to the baseline, ordered by mean LER. The blue bars indicate the model used for the experiments. (**a**) For 5 × 5*p*_*ij*_ DEM training, averaged across bases (*X* and *Z*) and the two cross-validation folds. The red horizontal line represents the performance of the finetuned ensemble. Error bars represent bootstrap standard errors from individual fidelities (499 resamples). (**b**) For 11 × 11 Pauli+ training. Error bars represent estimated mean error (N = 5). (**c**) Effect of ablations in performance and data efficiency. Decoding performance of the best performing subset of ablations for 5 × 5 DEM and 11 × 11 Pauli+. Colors indicate the number of training samples required for reaching performance parity with PyMatching for 11 × 11 Pauli+.

SimpleReadoutStack. We reduce the number of layers in the Readout ResNet from 16 to 1 (Extended Data Fig. 8b).

SimpleInputStack. We reduce the number of ResNet layers in the feature embedding from 2 to 1 (Extended Data Fig. 8b).

PoolingStabs. We do not scatter to two dimensions before pooling in the readout. The result is that we mean pool across all stabilizers instead of along data-qubit rows or columns (corresponding to logical observables).

NoAttBias. We remove the attention bias, both embedding and event indicator features. The Pauli+ experiments were done without attention bias.

NoNextStabPred. We remove the next stabilizer prediction loss from the loss.

FewerDims. We reduce the number of dimensions per stabilizer in the syndrome transformer to 30.

FewerLayers. We reduce the number of layers in the syndrome transformer from 3 to 1 for each round.

#### Input ablations

OnlyEvents. We only give syndrome information as detection events (removing measurements).

OnlyMeasurements. We only give syndrome information as raw qubit measurements (removing events).

For both of these, we point out that we give the cumulative sum (mod 2) of detection events as inputs where we are pretraining on a DEM (which does not provide the initial state of stabilizer measurements, and thus cannot be used to re-create absolute measurements).

## Online content

Any methods, additional references, Nature Portfolio reporting summaries, source data, extended data, supplementary information, acknowledgements, peer review information; details of author contributions and competing interests; and statements of data and code availability are available at 10.1038/s41586-024-08148-8.

## Supplementary information

Supplementary Information

## Figures and Tables

**Extended Data Table 1:**
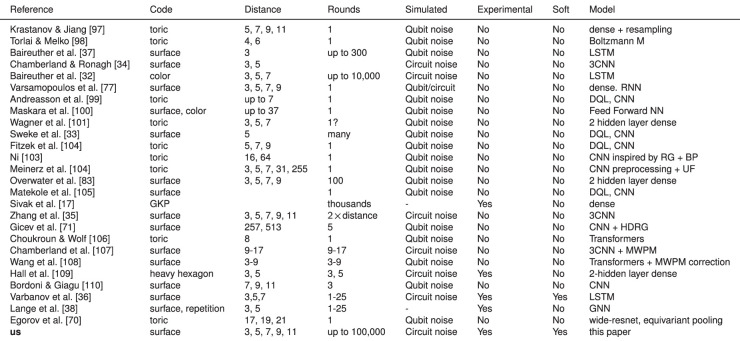
ML decoders, further references and details

Further references are refs. ^97–110^.

## Data Availability

The data for the Pauli+ simulations and documentation for loading the datasets are available at https://storage.mtls.cloud.google.com/gdm-qec. The data for Google’s Sycamore memory experiment are available at 10.5281/zenodo.6804040 (ref. ^42^).

## Code availability

Detailed pseudocode for all components of the neural-network decoding architecture is provided as part of Supplementary Information.
