# Supplementary material for: Learning high-accuracy error decoding for quantum processors
Source: Nature. 2024 Nov 20;635(8040):834–40. doi: 10.1038/s41586-024-08148-8 (PMC11602728; doi:10.1038/s41586-024-08148-8)
Supplement: Supplementary file 1 — Supplementary Information [file 41586_2024_8148_MOESM1_ESM.pdf]

nature portfolio

https://doi.org/10.1038/s41586-024-08148-8

Supplementary information

Learning high-accuracy error decoding for quantum processors

In the format provided by the authors and unedited

Supplementary information

Pseudocode

Algorithm 1: `AttentionWithBias`

/* Computes a single self-attention head. */

Input: $\boldsymbol{X} \in \mathbb{R}^{d_d \ell}$, vector representations of primary sequence. $\boldsymbol{B'} \in \mathbb{R}^{\ell \times \ell}$ attention bias.

Output: $\tilde{\boldsymbol{V}} \in \mathbb{R}^{d_{\text{mid}} \ell}$, updated representations of tokens in $\boldsymbol{X}$.

Parameters: $\mathcal{W}_{\boldsymbol{qkv}}$ consisting of: $\boldsymbol{W_q} \in \mathbb{R}^{d_{\text{attn}} d_d}$, $\boldsymbol{b_q} \in \mathbb{R}^{d_{\text{attn}}}$ $\boldsymbol{W_k} \in \mathbb{R}^{d_{\text{attn}} d_d}$, $\boldsymbol{b_k} \in \mathbb{R}^{d_{\text{attn}}}$
$\boldsymbol{W_v} \in \mathbb{R}^{d_{\text{mid}} d_d}$, $\boldsymbol{b_v} \in \mathbb{R}^{d_d}$.

$\boldsymbol{Q} \leftarrow \boldsymbol{W_q X} + \boldsymbol{b_q} \boldsymbol{1}^\intercal$ $\quad$ [[$\boldsymbol{Q}$uery $\in \mathbb{R}^{d_{\text{attn}} \ell}$]]
$\boldsymbol{K} \leftarrow \boldsymbol{W_k Z} + \boldsymbol{b_k} \boldsymbol{1}^\intercal$ $\quad$ [[$\boldsymbol{K}$ey $\in \mathbb{R}^{d_{\text{attn}} \ell}$]]
$\boldsymbol{V} \leftarrow \boldsymbol{W_v Z} + \boldsymbol{b_v} \boldsymbol{1}^\intercal$ $\quad$ [[$\boldsymbol{V}$alue $\in \mathbb{R}^{d_{\text{mid}} \ell}$]]
$\boldsymbol{S} \leftarrow \boldsymbol{K}^\intercal \boldsymbol{Q} + \boldsymbol{B'}$ $\quad$ [[$\boldsymbol{S}$core $\in \mathbb{R}^{\ell \ell}$]]
return $\tilde{\boldsymbol{V}} = \boldsymbol{V} \cdot \text{softmax}\left(\boldsymbol{S} / \sqrt{d_{\text{attn}}}\right)$

Algorithm 2: `MHAttentionWithBias`

/* Computes Multi-Head self-attention layer with attention bias. */

Input: $\boldsymbol{X} \in \mathbb{R}^{d_d \times \ell}$, vector representations sequence. $\boldsymbol{B} \in \mathbb{R}^{d_b \ell \ell}$ embedded attention bias.

Output: $\tilde{\boldsymbol{V}} \in \mathbb{R}^{d_d \ell}$, updated representations of tokens in $\boldsymbol{X}$.

Hyperparameters: H, number of attention heads

Parameters: $\mathcal{W}$ consisting of
For $h = 0, 1 \ldots H - 1$, $\mathcal{W}^h_{\boldsymbol{qkv}}$ consisting of:
| $\boldsymbol{W}^h_{\boldsymbol{q}} \in \mathbb{R}^{d_{\text{attn}} d_d}$, $\boldsymbol{b}^h_{\boldsymbol{q}} \in \mathbb{R}^{d_{\text{attn}}}$,
| $\boldsymbol{W}^h_{\boldsymbol{k}} \in \mathbb{R}^{d_{\text{attn}} d_d}$, $\boldsymbol{b}^h_{\boldsymbol{k}} \in \mathbb{R}^{d_{\text{attn}}}$,
| $\boldsymbol{W}^h_{\boldsymbol{v}} \in \mathbb{R}^{d_{\text{mid}} d_d}$, $\boldsymbol{b}^h_{\boldsymbol{v}} \in \mathbb{R}^{d_{\text{mid}}}$.
$\boldsymbol{W_b} \in \mathbb{R}^{h \times d_b}$
$\boldsymbol{W_o} \in \mathbb{R}^{d_d d_{\text{mid}}}$, $\boldsymbol{b_o} \in \mathbb{R}^{d_{\text{out}}}$.

$\boldsymbol{B'} \leftarrow \boldsymbol{W_b B}$
for $h = 0, 1 \ldots H - 1$ **do**
| $\boldsymbol{Y}^h \leftarrow$ `AttentionWithBias`$(\boldsymbol{X}, \boldsymbol{B'}[h, :, :] | \mathcal{W}^h_{\boldsymbol{qkv}})$
end
$\boldsymbol{Y} \leftarrow [\boldsymbol{Y}^0; \boldsymbol{Y}^1; \ldots; \boldsymbol{Y}^{H-1}]$
return $\tilde{\boldsymbol{V}} = \boldsymbol{W_o Y} + \boldsymbol{b_o} \boldsymbol{1}^\intercal$

Algorithm 3: `GatedDenseBlock`

Input: $\boldsymbol{X} \in \mathbb{R}^{d_d \times \ell}$, input
Output: $\boldsymbol{Y} \in \mathbb{R}^{d_d \times \ell}$, output
Parameters: $\boldsymbol{W}_1 \in \mathbb{R}^{wd_d \times d_d}, \boldsymbol{W}_2 \in \mathbb{R}^{d_d \times (wd_d/2)}, \boldsymbol{b}_1 \in \mathbb{R}^{wd_d}, \boldsymbol{b}_2 \in \mathbb{R}^{d_d}$
Hyperparameters: w, widening factor

for $l = 0, 1, \ldots, \ell - 1$ **do**
  $\boldsymbol{Y} \leftarrow \boldsymbol{W}_1 \boldsymbol{X}[:, l] + \boldsymbol{b}_1 \mathbf{1}^\intercal$
  $\boldsymbol{Y} \leftarrow \texttt{GELU}(\boldsymbol{Y}[: (wd_d/2)])\boldsymbol{Y}[(wd_d/2) :]$
  $\boldsymbol{Y}^l \leftarrow \boldsymbol{W}_2 \boldsymbol{Y} + \boldsymbol{b}_2 \mathbf{1}^\intercal$
end
return $\boldsymbol{Y} = [\boldsymbol{Y}^0; \boldsymbol{Y}^1; \ldots; \boldsymbol{Y}^{\ell-1}]$

Algorithm 4: `ScatteringResidualConvBlock`

Input: $\boldsymbol{X} \in \mathbb{R}^{d_d \times \ell}$
Output: $\boldsymbol{Z} \in \mathbb{R}^{d_d \times \ell}$
Hyperparameters: ℓ, d number of stabilizers and code distance, related by $\ell = d^2 - 1$. L number of CNN layers. $c \in \mathbb{R}^L$ output channels of CNN.

/* Scatter to 2D */
for $coord = (0, 0), (0, 1) \ldots (d, d)$ **do**
  if $\exists$ *stabilizer index with coordinates coord* **then** $\boldsymbol{Y}[coord, :] \leftarrow \boldsymbol{X}[:, index]$
  else $\boldsymbol{Y}[coord, :] \leftarrow \boldsymbol{P}$
end
for $l = 0, 1, \ldots, L - 1$ **do**
  /* Dilated convolutions */
  $\tilde{\boldsymbol{Y}} \leftarrow \texttt{layer\_norm}(\boldsymbol{Y})$
  $\tilde{\boldsymbol{Y}} \leftarrow \texttt{GELU}(\texttt{CNN}(\tilde{\boldsymbol{Y}}; \text{kernel\_size} = 3, \text{channels} = c[l]))$
  if $c[l] \neq d_d$ **then**
    $\tilde{\boldsymbol{Y}} \leftarrow \texttt{CNN}(\tilde{\boldsymbol{X}}; \text{kernel\_size} = 1, \text{channels} = d_d)$
  end
  $\boldsymbol{Y} \leftarrow \boldsymbol{Y} + \tilde{\boldsymbol{Y}}$ $[\![\boldsymbol{Y} \in \mathbb{R}^{(d+1)(d+1)d_d}]\!]$
end
/* Gather from 2D */
for $coord = (0, 0), (0, 1) \ldots (d, d)$ **do**
  if $\exists$ *stabilizer index with coordinates coord* **then**
    $\boldsymbol{Z}[:, index] \leftarrow \boldsymbol{Y}[coord, :]$
  end
end
return $\boldsymbol{Z}$

Algorithm 5: `RNNCore`

Input: $\boldsymbol{X} \in \mathbb{R}^{d_d \times \ell}$, decoder state. $\boldsymbol{S} \in \mathbb{R}^{d_d \times \ell}$, embedded stabilizers at current round.
$\boldsymbol{B} \in \mathbb{R}^{d_b \ell \ell}$ embedded attention bias.

Output: $\boldsymbol{X} \in \mathbb{R}^{d_d \times \ell}$, updated decoder state.

Hyperparameters: $L \in \mathbb{N}$, number of layers

$\boldsymbol{X} \leftarrow (\boldsymbol{X} + \boldsymbol{S})/\sqrt{2}$
for $l = 0, 1, \ldots, L - 1$ **do**
  /* SyndromeTransformer */
  $\tilde{\boldsymbol{X}} \leftarrow$ `layer_norm`$(\boldsymbol{X})$
  $\boldsymbol{X} \leftarrow \boldsymbol{X} +$ `MHAttentionWithBias`$(\tilde{\boldsymbol{X}}, \boldsymbol{B})$
  $\tilde{\boldsymbol{X}} \leftarrow$ `layer_norm`$(\boldsymbol{X})$
  $\boldsymbol{X} \leftarrow \boldsymbol{X} +$ `GatedDenseBlock`$(\tilde{\boldsymbol{X}})$
  $\boldsymbol{X} \leftarrow$ `ScatteringResidualConvBlock`$(\boldsymbol{X})$
end
return $\boldsymbol{X}$
